# Extracellular vesicle-mediated amyloid transfer to neural progenitor cells: implications for RAGE and HIV infection

**DOI:** 10.1186/s13041-020-0562-0

**Published:** 2020-02-17

**Authors:** Ibolya E. András, Marta Garcia-Contreras, Christopher Yanick, Paola Perez, Brice Sewell, Leonardo Durand, Michal Toborek

**Affiliations:** 1 Department of Biochemistry and Molecular Biology, University of Miami School of Medicine, 1011 NW 15th Street, Gautier Building, Room 528, Miami, FL 33136-1019 USA; 2 Diabetes Research Institute, University of Miami School of Medicine, 1450 NW 10th Ave, Miami, FL 33136-1011 USA

**Keywords:** Extracellular vesicles, Blood-brain barrier, Amyloid beta, Neural progenitor cells, RAGE

## Abstract

Amyloid beta (Aβ) deposition was demonstrated to be elevated in the brains of HIV-infected patients and associated with neurocognitive decline; however, the mechanisms of these processes are poorly understood. The goal of the current study was to address the hypothesis that Aβ can be transferred via extracellular vesicles (ECVs) from brain endothelial cells to neural progenitor cells (NPCs) and that this process can contribute to abnormal NPC differentiation. Mechanistically, we focused on the role of the receptor for advanced glycation end products (RAGE) and activation of the inflammasome in these events. ECVs loaded with Aβ (Aβ-ECVs) were readily taken up by NPCs and Aβ partly colocalized with the inflammasome markers ASC and NLRP3 in the nuclei of the recipient NPCs. This colocalization was affected by HIV and RAGE inhibition by a high-affinity specific inhibitor FPS-ZM1. Blocking RAGE resulted also in an increase in ECV number produced by brain endothelial cells, decreased Aβ content in ECVs, and diminished Aβ-ECVs transfer to NPC nuclei. Interestingly, both Aβ-ECVs and RAGE inhibition altered NPC differentiation. Overall, these data indicate that RAGE inhibition affects brain endothelial ECV release and Aβ-ECVs transfer to NPCs. These events may modulate ECV-mediated amyloid pathology in the HIV-infected brain and contribute to the development of HIV-associated neurocognitive disorders.

## Introduction

HIV-infected brains were shown to have increased amyloid beta (Aβ) deposition [1–6]. This phenomenon has been linked to the development of cognitive dysfunction based on the observation that early beta-amyloidosis in HIV-infected patients was associated with HIV-associated neurocognitive disorders (HAND) [3, 7]. Aβ deposition occurs mostly in the perivascular space [3, 7–9], which points to the brain microvessels having a role in amyloid pathology. In support of this notion, the blood-brain barrier (BBB), a critical player in the brain infection by HIV and the development of HIV-associated cerebrovascular comorbidities [10, 11], was postulated to regulate Aβ homeostasis as an interface contributing to Aβ accumulation in the brain [12]. Indeed, it was demonstrated that the receptor for advanced glycation end products (RAGE) can mediate Aβ transport across the BBB and accumulation in the brain [13]. Similarly, RAGE was shown to be involved in HIV-induced accumulation of Aβ in brain endothelial cells, a structural component of the BBB [14].

Extracellular vesicles (ECVs), such as exosomes, were demonstrated before to be important in HIV and Aβ pathology [15–21]. We observed that HIV increased the shedding of ECVs carrying Aβ from brain endothelial cells. Moreover, brain endothelial cell-derived ECVs transferred Aβ to cells of the neurovascular unit, namely to astrocytes and pericytes [22], prompting us to hypothesize that a similar process may also increase Aβ exposure of other cells found in close proximity of the brain microvessels, including the neural progenitor cells (NPCs). In fact, ~ 47% of dividing progenitor and 46% of transit amplifying cells (i.e., cells that give rise to neuroblasts) are located within 5 μm of the endothelium [23, 24].

In this work we aimed to evaluate possible mechanisms involved in shedding of ECVs by brain endothelial cells and Aβ-ECVs transfer to NPCs. Because Aβ-ECVs may affect neurogenesis [25], we also focused on the impact of this process on differentiation of NPCs into neurons. The importance of this line of experimentation is related to the notion that aberrant NPC differentiation and neurogenesis may contribute, at least in part, to the cognitive deficits observed in HIV-infected patients [26].

Based on the observations that a) HIV can increase RAGE expression in brain endothelial cells [14], b) HIV-induces Aβ accumulation in brain endothelial cells via a RAGE-dependent mechanism [14], and c) RAGE may be involved in microvesicle secretion [27], we hypothesize in the current study that RAGE may be a key player in the HIV-induced brain endothelial ECV release and Aβ-ECVs transfer to NPCs. In addition, because both HIV infection [28] and Aβ pathology [29, 30] were linked to the inflammasome pathway, and RAGE was shown to signal through the NLR family pyrin domain containing 3 (NLRP3) inflammasome [31], we further aimed to examine the impact of Aβ-ECV transfer on the NLRP3 inflammasome in NPCs.

## Materials and methods

### Cell cultures

#### Human brain microvascular endothelial cells (HBMEC)

HBMEC used in the present study represent a stable, well characterized, and differentiated human brain endothelial cell line [32]. Briefly, normal human brain endothelial cells were transduced by lentiviral vectors incorporating human telomerase or SV40T antigen. Among several stable immortalized clones obtained by sequential limiting dilution cloning of the transduced cells, the hCMEC/D3 cell line (referred here as HBMEC) was selected as expressing normal endothelial markers and demonstrating blood–brain barrier characteristics. HBMEC for the present study were supplied by Dr. Couraud (Institut Cochin, Paris, France). HBMEC were cultured on collagen type I (BD Biosciences Pharmingen, San Jose, CA) coated dishes in EBM-2 medium (Clonetics, East Rutherford, NJ) supplemented with VEGF, IGF-1, EGF, basic FGF, hydrocortisone, ascorbate, gentamycin and 0.5% exosome depleted fetal bovine serum (Exo-FBS; System Biosciences, Mountain View, CA).

#### Human neural progenitor cells (NPCs)

An immortalized NPC line ReNcell VM, derived from 10-week human ventral mesencephalon, was obtained from Millipore and cultured according to the manufacturer’s protocols. The cells were validated for high expression of Sox2 and nestin as well as for their self-renewal and differentiation capacity. Cells were grown on laminin coated tissue culture dishes in a maintenance medium (Millipore), containing 20 ng/ml FGF-2 and 20 ng/ml of rhEGF. Cells were used for experiments at < 80% confluence, 3 days after plating.

### HIV infection and treatment factors

HIV stock was generated using human embryonic kidney (HEK) 293 T cells (ATCC) transfected with pYK-JRCSF plasmid containing full-length proviral DNA. Throughout the study, HBMEC were exposed to HIV particles at the p24 level of 30 ng/ml as previously reported [33]. Treatment was terminated by removing cell culture media containing HIV, followed by washing the cells in PBS.

Aβ (1–40) and Aβ (1–40) HiLyte were purchased from Anaspec (San Jose, CA) and Aβ (1–40) was dissolved in PBS. Freshly solubilized Aβ solutions without pre-aggregation were used for experiments as such a form of Aβ was demonstrated to induce proinflammatory reactions in isolated rat brain microvessels [34]. Aβ (1–40) HiLyte was dissolved first in a basic buffer (0.1 M NH_4_OH) and then diluted further in PBS as suggested by the manufacturer. Cells were treated with Aβ (1–40) or Aβ (1–40) HiLyte at the concentration of 100 nM in complete medium.

FPS-ZM1 (FPS, Cayman Chemicals, Ann Arbor, MI, USA) is a high-affinity RAGE-specific inhibitor [35]. 1 mM stock solution was prepared in DMSO. Cells were pretreated with 500 nM FPS for 2 h followed by coexposure with HIV and/or 100 nM Aβ (1–40).

GW 4869 (Millipore Sigma, Burlington, MA, USA) is a cell-permeable, non-competitive inhibitor of neutral sphingomyelinases. 5 mM stock solution of GW4869 was prepared in DMSO. Because of low solubility, the stock was incubated at 37 °C for 15 min and supplemented with 1/20 volume of 5% methane-sulfonic acid (MSA) before adding GW4869 to cell culture medium [36]. Control cultures were exposed to vehicle, i.e., 0.4% DMSO supplemented with 1/20 volume of 5% MSA. Cells were pretreated with the solubilized GW4869 (20 μM) for 1 h followed by coexposure with HIV and/or 100 nM Aβ (1–40).

### Treatment of brain endothelial cells and ECV isolation

Confluent HBMEC were exposed to HIV and/or Aβ (1–40)/Aβ (1–40) HiLyte for 48 h. ECVs were isolated from conditioned medium using ExoQuick-TC exosome precipitation solution (System Biosciences) according to the manufacturer’s specifications. Briefly, 10 ml culture medium (from 1.7 × 10^7^ cells at confluency cultured in a P100 dish) was centrifuged at 3000 g for 15 min to remove cells and debris. Then, the samples were mixed thoroughly with 2 ml of Exo-Quick exosome precipitation solution and incubated overnight at 4 °C. The next day, the samples were centrifuged at 1500 g for 30 min, the supernatants were removed and centrifuged again at 1500 g for 5 min. The ECV pellets were resuspended in 400 μl PBS and used for further studies. The aliquots of 20 μl of ECV suspension for every 100 μl of cell culture media was used for NPC treatment.

### Nanoparticle tracking analysis (NTA)

ECVs were analyzed by NanoSight model NS300 (Malvern Instruments Company, Nanosight, Malvern, United Kingdom) as described earlier [22]. Briefly, ECV pellet samples obtained in the process of ECV isolation were resuspended in 100 μl 4% paraformaldehyde-PBS and further diluted 100 fold in PBS for analysis. Three 90 s videos were recorded for each sample. The obtained data were analyzed using Nanosight NTA 2.3 Analytical Software (Malvern Instruments Company) with the detection threshold optimized for each sample and screen gain at 10 to track the maximal number of particles with minimal background. Most of isolated ECVs carry fluorescent Aβ (1–40) cargo. In addition, Aβ is associated with ECVs of different size (see Additional file 1: Figure S1).

### Protein isolation and western blot

Proteins from NPCs were extracted with Radio Immuno Precipitation Assay (RIPA) buffer (Pierce, IL, USA) with freshly added protease inhibitors and 1% Triton-X 100 to inactivate HIV. ECV protein concentration was measured by BCA protein assay kit (Pierce). Equal amount of proteins (8–16 μg/lane) was loaded on sodium dodecyl sulfate polyacrylamide 4–20% ready gels (BioRad, Hercules, CA) and electrotransferred to a nitrocellulose membrane using a transfer pack system (BioRad). The blots were probed at 4 °C with rabbit anti-ASC antibody (Catalog # AL177, Adipogen Life Sciences, San Diego, CA), rabbit anti-NLRP3 antibody (Catalog # LSB4321, LSBio, Seattle, WA) or mouse anti-caspase-1 antibody (Catalog # sc-514, Santa Cruz Biotechnology Inc) (1:400) in 5% milk-TBS-T. After washing 3 times with TBS-T, the samples were incubated with secondary antibodies diluted at 1:20,000 (anti-mouse 800CW or anti-rabbit 680RD). Anti-GAPDH antibody conjugated with Dylight 680 (1:20,000; Novus Biologicals, Littleton, CO, USA) was employed as an internal control. Signal detection was performed using Image Studio 4.0 software (LI-COR). In our cells, the 16.5 kDa ASC band corresponded to a splice variant according to the manufacturer. For NLRP3, additional bands were found above and below the manufacturer-predicted 101, 114 kDa bands. For caspase-1 a very weak band of ~ 40 kDa was found (see Additional file 1: Figure S3).

### Caspase-1 activity assay

Caspase-1 activity was measured after a 5 h and 24 h exposure to ECVs with the Caspase-Glo 1 Inflammasome assay (Catalog # G9951, Promega, Madison, WI) following the manufacturer’s instructions.

### Enzyme linked Immunosorbent assay (ELISA)

ELISA was used to determine levels of total human Aβ (1–40) (R&D Systems, Minneapolis, MN, USA) in the isolated brain endothelial ECVs. IL-1β, VEGF-A and BDNF levels were also measured by ELISA from the cell culture medium after 3 days of NPC differentiation (R & D Systems).

### Fluorescence microscopy

NPCs (28,000 cells/well) were cultured and treated on laminin coated 8-chambered glass slides (ibidi USA, Madison, WI). Then, the cells were washed with PBS and fixed with absolute ethanol for 30 min at 4 °C. After washing with PBS, nuclei were stained with DRAQ5 for 5 min followed by PBS wash. Slides were mounted using ProLong Gold antifade reagent (Life Technologies). Green fluorescence (originating from Aβ HiLyte Alexa Fluor488) and red fluorescence (from DRAQ5) were acquired directly using a Nikon Eclipse Ti-U fluorescence microscope.

### Confocal microscopy

ECV-treated NPCs cultured on laminin coated chambered glass slides (Becton Dickinson Biosciences, San Jose, CA) were fixed with ethanol for 30 min at 4 °C. After washing with PBS and blocking with 3% bovine serum albumin in PBS for 30 min, samples were incubated overnight at 4 °C with the primary antibody: rabbit anti-ASC polyclonal antibody (Catalog # AL177, 1:400), rabbit anti-NLRP3 polyclonal antibody (Catalog # LSB4321, 1:400), mouse anti-Hu C/D monoclonal antibody (Catalog # A-21271, 1:300) (from Invitrogen, Carlsbad, CA), rabbit anti-NeuN monoclonal antibody (Catalog # 24307, 1:200, Cell Signaling Technology, Danvers, MA) or mouse anti-doublecortin (DCX) monoclonal antibody (Catalog # GTX60612, 1:200, GeneTex, Irvine, CA). Then, the excess of primary antibody was removed, slides were washed with PBS, and incubated with Alexa Fluor488/594-conjugated secondary antibody (1:200, Invitrogen) for 2 h at room temperature. In some experiments, nuclei were stained with DRAQ5 followed by PBS wash. Slides were mounted using ProLong Gold Antifade reagent with or without 4′,6-diamidino-2-phenylindole (DAPI, Invitrogen) to visualize the nuclei. Specimens were covered with coverslips and the immunofluorescent images were evaluated and captured under confocal microscopy. Red fluorescence originating from DRAQ5, blue fluorescence from DAPI, and green fluorescence from ECV-Aβ HiLyte-Alexa Fluor488 was acquired directly using confocal microscopy (Olympus, Fluoview 1200, dry lens UPLFLN 40x NA: 0.75 or 60x oil immersion lens, room temperature) and did not require the use of primary or secondary antibody.

For Aβ measurements, fields were chosen at random and acquisition and quantification were performed using the FV10-ASW3.1 software. For the nuclear Aβ measurements, mean fluorescence intensity was measured in the nuclear areas outlined by the DRAQ5 or DAPI staining. For ASC and NLRP3 measurements, the total area fluorescence intensity on the acquired images was normalized to the number of nuclei. For the nuclear ASC, NLRP3 measurements, mean fluorescence intensity was measured in random nuclear areas outlined by the DAPI staining.

### NPC differentiation

NPCs were seeded on laminin coated 8-well chambered glass slides (28,000 cells/well) and incubated overnight at 37 °C in maintenance culture medium (Millipore), containing 20 ng/ml FGF-2 and 20 ng/ml of rhEGF. The following day, the medium was changed to maintenance medium without growth factors to induce differentiation. Cells were allowed to differentiate for 3 days in the presence or absence of the employed ECVs. To block RAGE signaling, selected NPC cultures were pretreated with 500 nM FPS for 2 h, followed by coexposure with ECVs for 3 days. After 3 days, cells were washed with PBS, fixed with ethanol for 30 min at 4 °C, and subjected to immunostaining for Hu C/D, NeuN, and DCX markers to assess neurons at different stages of development. Fluorescence images were acquired randomly through a confocal laser-scanning microscope (Olympus Fluoview 1200) and analyzed using FV10-ASW3.1 software. Hu C/D, NeuN positive and NeuN/DCX double positive cells were counted and expressed as percentage of the total cell number.

### Luminex MagPix assay

Cell culture media samples collected after 3 days of differentiation were analyzed for cytokine/chemokine panel (G-CSF, IL-4, CCL2, Fractalkine, PDGF-AA, PDGF-AB/BB, VEGF-A, BDNF, NGF-β; Milliplex MAP Human Cytokine/Chemokine Magnetic Bead Panel, Millipore Corp., Billerica, MA) following the kit-specific protocols provided by Millipore. Analytes were quantified using a Magpix analytical test instrument, which utilizes xMAP technology (Luminex Corp., Austin, TX), and xPONENT 4.1 software (Luminex). xMAP technology uses fluorescent-coded magnetic microspheres coated with analyte-specific capture antibodies to simultaneously measure multiple analytes in a specimen. After micro-spheres have captured the analytes, a biotinylated detection antibody binds to that complex. Streptavidin PE then attaches as a reporter molecule. Inside the instrument, magnetic beads are held in a monolayer by a magnet, where two LEDs are used to excite the internal micro-sphere dye and the dye of the reporter molecule, respectively. A CCD camera captures these images, which are then analyzed by Milliplex Analyst software (Millipore).

Concentrations of cytokines (pg/ml) were determined on the basis of the fit of a standard curve for mean fluorescence intensity versus pg/ml. Two quality controls were run with each assay (control 1, low level; control 2, high level.) All cytokines were found to fall within the quality control ranges except for VEGF-A. Therefore VEGF-A levels were separately determined with ELISA.

### Statistical analysis

Data were analyzed using GraphPad Prism 6.0 (Graphpad Software, San Diego, CA). ANOVA was used to compare responses among treatments. Treatment means were compared using All Pairwise Multiple Comparison Procedures and *p* < 0.05 was considered significant.

## Results

### RAGE regulates generation of ECVs by HBMEC and their Aβ levels

ECVs were demonstrated to be involved in HIV and Aβ pathology [15–21, 37], and in intercellular transfer of Aβ [22]. In addition, the RAGE pathway may both interfere with ECV release, as it was shown in macrophages [27], and with Aβ accumulation in brain endothelial cells [14]. Therefore, we evaluated the involvement of RAGE in brain endothelial ECV secretion and regulation of their Aβ cargo load. HBMEC were exposed to 30 ng/ml HIV particles and/or 100 nM Aβ (1–40) for 48 h. Selected HBMEC cultures were pretreated with 500 nM FPS-ZM1 (FPS, a specific RAGE inhibitor) for 2 h, followed by co-treatment with 30 ng/ml HIV particles and/or 100 nM Aβ (1–40) for 48 h. ECVs released by HBMEC to the culture media were isolated and characterized for their number, size, and Aβ content in the subsequent series of experiments.

**Fig. 1:**
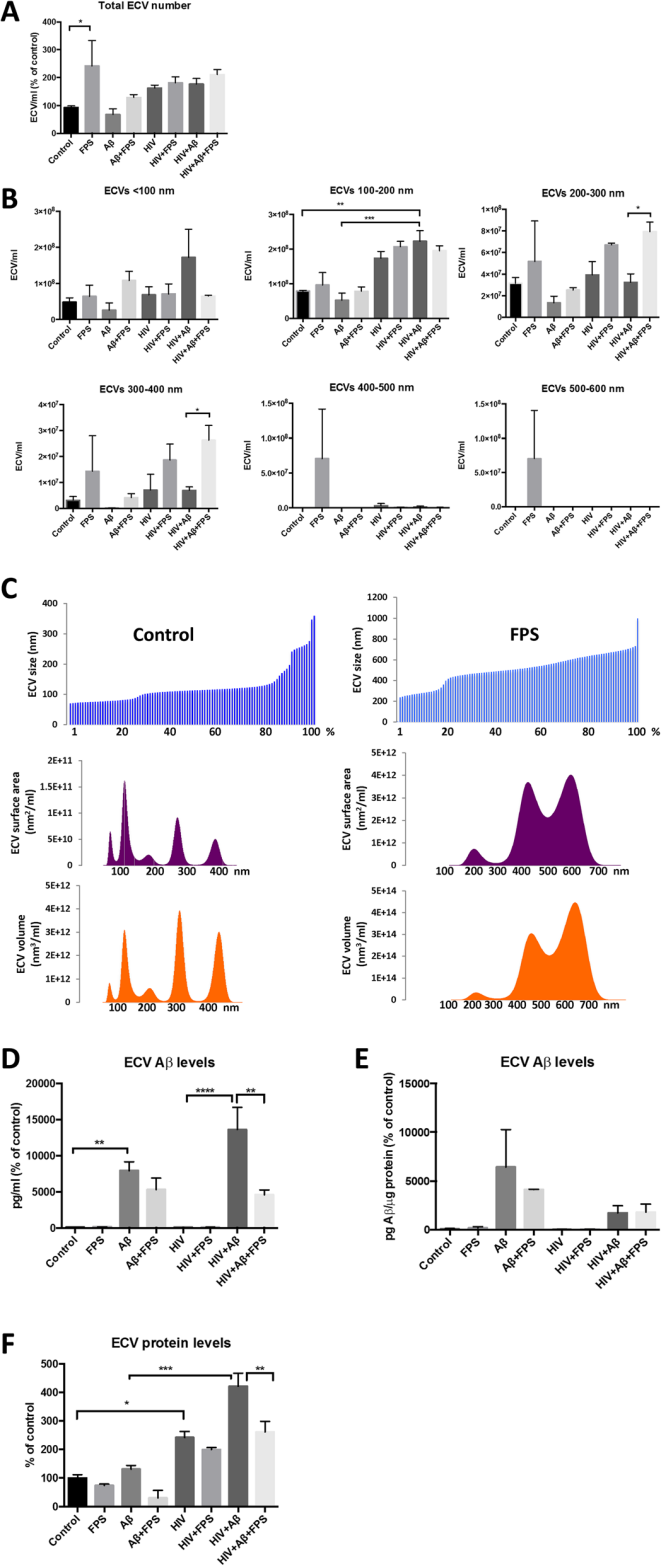
Release of brain endothelial ECVs and their Aβ levels. HBMEC were exposed to 30 ng/ml HIV particles and/or 100 nM Aβ (1–40) for 48 h. Selected cultures were pretreated with 500 nM FPS-ZM1 (FPS) for 2 h followed by cotreatment with 30 ng/ml HIV particles and/or 100 nM Aβ (1–40) for 48 h. ECVs were isolated from the culture media. **a** Total ECV number as measured by nanoparticle tracking analysis (NTA). Values are mean ± SEM; *n* = 3–4. **b** ECV number according to their size distribution and measured as in A. Values are mean ± SEM; n = 3–4. **c** Size, surface area, and volume distribution of ECVs shed by a representative control and FPS-treated HBMEC. **d** & **e** ECV Aβ (1–40) levels as measured by ELISA and normalized either to (**d**) media volume or (**e**) ECV protein levels. Values are mean ± SEM; *n* = 5–7. **f** ECV protein levels as measured by the BCA assay. Values are mean ± SEM; n = 3–4. Statistically significant at *p < 0.05, **p < 0.01, or ***p < 0.001

Nanoparticle tracking analysis (NTA) was employed to quantify the number of ECVs produced by HBMEC in response to HIV, Aβ, and/or FPS. Compared to the control, treatment with FPS resulted in a significantly higher total ECV number in the media of treated HBMEC (Fig. 1a). While the majority of the remaining experimental groups showed a tendency to increase ECV levels as compared to controls, the changes were not significant. We then analyzed the impact of HIV and/or Aβ on ECV levels grouped according to their dimensions, ranging from < 100–600 nm (Fig. 1b). Exposure of HBMEC to HIV plus Aβ significantly elevated levels of 100–200 nm sized ECVs in the media as compared to the Aβ and the control groups. Similar tendency was observed for smaller (below 100 nm) ECVs; however, the differences did not reach statistical significance. These groups of ECVs appear to be particularly important because their size corresponds to exosomes. Interestingly, a co-treatment with RAGE inhibitor increased the number of slightly larger ECVs, i.e., 200–300 nm and 300–400 nm ECVs in the HIV + Aβ + FPS group as compared to the HIV + Aβ group. Treatment with FPS alone had a tendency to increase levels of ECVs of larger sizes, such as 400–600 nm in dimension. While these effects were not statistically significant due to a high standard deviation, treatment with FPS alone profoundly affected the overall ECV size, surface area, and volume distribution as illustrated for the representative control and FPS samples (Fig. 1c).

We then analyzed the involvement of RAGE in the modulation of Aβ cargo load in HBMEC-derived ECVs. Using the total population of ECVs from Fig. 1a, Aβ (1–40) levels in ECV lysates were measured by ELISA (Fig. 1d and e). When normalized to the media volume, exposure of HBMEC to 100 nM Aβ (1–40) markedly increased Aβ cargo load in ECVs. This effect was more pronounced by a co-exposure to HIV. Interestingly, Aβ (1–40) levels were significantly downregulated in the HIV + Aβ-ECVs+FPS as compared to the HIV + Aβ-ECVs (Fig. 1d). This FPS-induced effect changed when Aβ-ECV levels were normalized not to the media volume but to ECV protein levels (Fig. 1e). Using this normalization approach, coexposure to FPS had a tendency to decrease Aβ cargo in the Aβ + FPS ECVs, but the changes were not statistically significant. At the same time, FPS had no impact on Aβ levels in the HIV + Aβ + FPS ECVs. The reason for the observed differences was a substantial impact of the employed treatment factors, including FPS, on the overall protein levels in ECVs. As shown in Fig. 1f, treatment with FPS significantly reduced protein levels in ECVs in the HIV + Aβ + FPS group as compared to the HIV + Aβ group. In addition, a trend of decreasing protein load was noted in FPS-ECVs as compared to controls, and in the Aβ + FPS-ECVs as compared to Aβ-ECVs (Fig. 1f). At the same time, protein levels in the parent cells remained at the control levels and were not affected by the employed treatment factors (Additional file 1: Figure S2F). Overall, the results in Fig. 1 indicate that ECVs contain Aβ as part of their cargo load. It also appears that RAGE has dual impact on this process, influencing both ECV number/size and their Aβ cargo. Indeed, treatment with FPS tends to decrease the total ECV protein cargo, while increasing ECV number and size.

In addition to RAGE, neutral sphingomyelinase (nSMase) was reported to be involved in ECV generation [38]. Therefore, we evaluated the impact of nSMase inhibition with GW4869 on production of ECVs by HBMEC and their Aβ load. Blocking nSMase did not significantly affect ECVs production by HBMEC. When normalized to the media volume, GW4869 significantly decreased Aβ levels in Aβ- and HIV + Aβ-ECVs. However, these effects were not observed when the results were normalized to ECV protein levels (Additional file 1: Figure S2).

### RAGE affects the uptake of HIV + Aβ-ECVs by neural progenitor cells (NPCs)

ECVs shed by HBMEC can effectively transfer their cargo to the neighboring cells. Indeed, we demonstrated that HBMEC-derived ECVs can deliver Aβ to astrocytes and pericytes [22], i.e., the cells of the neurovascular unit [39]. This process may also increase Aβ exposure of other cells found in a close proximity to the brain microvessels, including NPCs located in neurogenic niches in the perivascular space [24]. Therefore, we next investigated whether the regulatory role of RAGE on the generation of ECVs by HBMEC and their Aβ cargo can also impact Aβ transfer to NPCs. In support of this line of investigation, RAGE was demonstrated to be expressed in adult hippocampal NPCs [25].

**Fig. 2:**
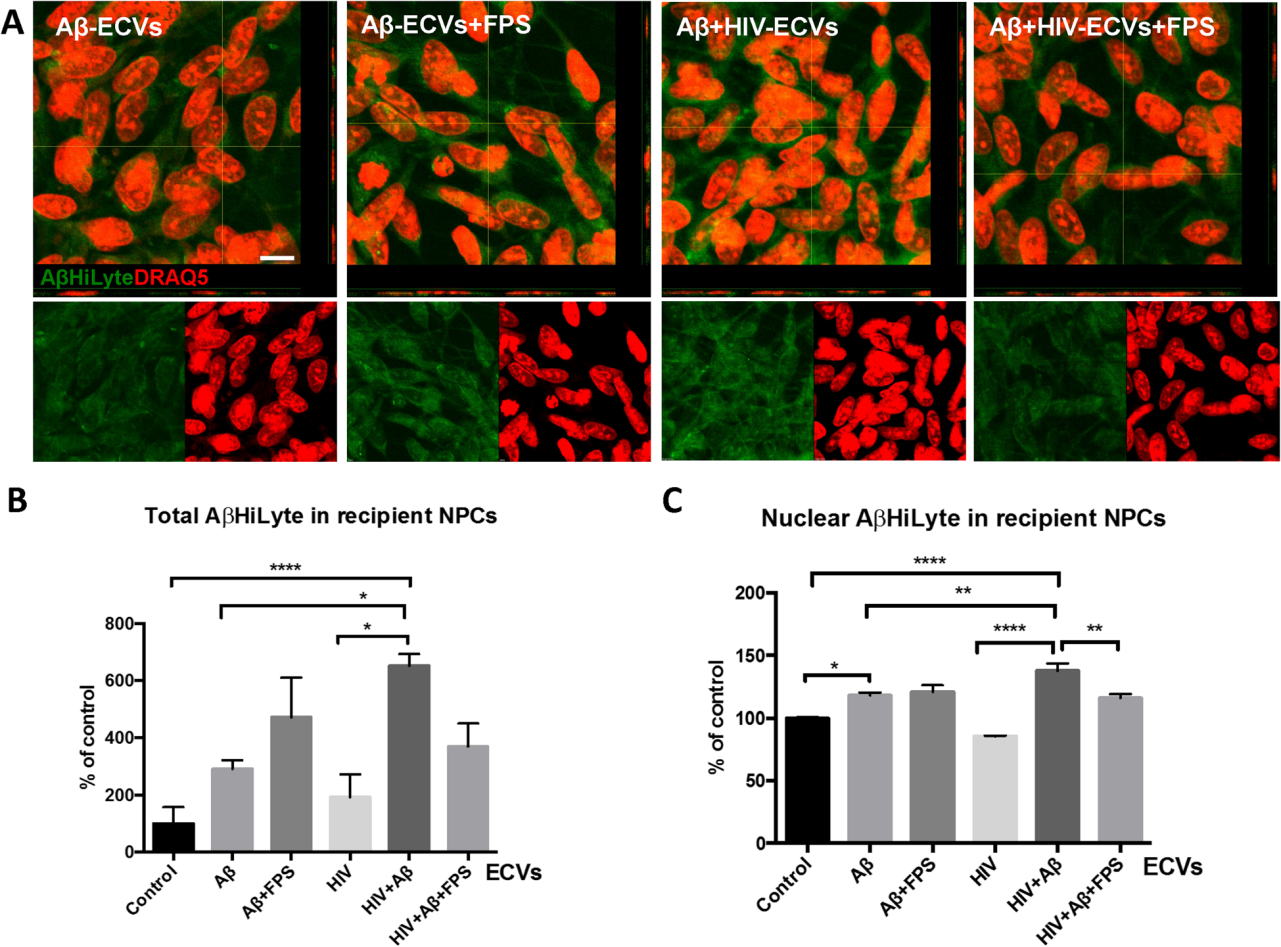
Uptake of ECVs by NPCs. HBMEC were treated with HIV and/or Aβ and ECVs were isolated as in Fig. 1; however, Aβ (1–40) HiLyte was used instead of non-florescent Aβ (1–40). Then, human NPCs were exposed to HBMEC-derived ECVs for 24 h, with selected cultures pretreated with 500 nM FPS-ZM1 (FPS) for 2 h followed by cotreatment with the isolated ECVs. Images were performed by confocal microscopy z-stacking. **a** Transferred Aβ (1–40) HiLyte (green) from brain endothelial ECVs to NPCs. Representative images from three experiments. DRAQ5 staining (red) visualizes the NPC nuclei. Scale bar: 10 μm. **b** Quantification of total Aβ (1–40) HiLyte fluorescence in recipient NPCs from the confocal images. Values are mean ± SEM; *n* = 8–11. **c** Quantification of nuclear Aβ (1–40) HiLyte fluorescence in recipient NPCs from the confocal images. Values are mean ± SEM; *n* = 47–86 from randomly selected images. Statistically significant at *p < 0.05, **p < 0.01, or ****p < 0.0001

HBMEC were treated with Aβ HiLyte and/or HIV, followed by isolation of ECVs as in Fig. 1. Then, human NPCs were exposed to these ECVs for 24 h. Selected NPC cultures were also pretreated for 2 h with 500 nM FPS, followed by cotreatment with ECVs for 24 h. Transfer of Aβ from ECVs to recipient NPCs was assessed by measuring fluorescence intensity of Aβ HiLyte (green) by confocal microscopy (Fig. 2a). NPC nuclei were stained with DRAQ5 (red). Quantification of the results revealed that the highest Aβ uptake was observed in NPC cultures exposed to ECVs derived from HIV plus Aβ-treated HBMEC. This effect was statistically higher as compared to cultures treated alone with Aβ-ECVs and HIV-ECVs (Fig. 2b). Similar results were obtained when quantifying Aβ uptake to the nuclei of the recipient NPCs (Fig. 2c). RAGE inhibition with FPS on the recipient NPCs significantly reduced nuclear transfer of Aβ by Aβ + HIV ECVs and exhibited a strong tendency to decrease the total cellular transfer of Aβ by the same ECVs.

### ECV-mediated Aβ transfer to NPCs is associated with inflammasome protein changes

Both Aβ pathology and HIV infection have been linked to the induction of the inflammasome pathway [28, 30]. Therefore, we explored the impact of HBMEC-derived Aβ-ECVs and HIV + Aβ-ECVs on the inflammasome pathway in the recipient NPCs. These experiments were performed without priming NPCs with LPS, as it is frequently done in the literature, so as not to mask the impact of the employed treatment factors. In addition, it was shown before that HIV Tat protein can prime and induce the NLRP3 inflammasome pathway [40]. HBMEC were treated, and ECVs were isolated as in Figs. 1 and 2, followed by exposure to NPCs for 24 h. Similar to Fig. 2, selected NPC cultures were pretreated with 500 nM FPS for 2 h to inhibit RAGE. The experiments focused on the NLRP3, a major pattern recognition receptor that is expressed in response to a variety of stimuli, including Aβ and HIV [28, 30], and on the adaptor protein called apoptosis-associated speck-like protein containing CARD (ASC), both critical mediators of the inflammasome pathway. We visualized the cellular distribution and the complex cellular/nuclear pattern of both NLRP3 and ASC by confocal microscopy.

**Fig. 3:**
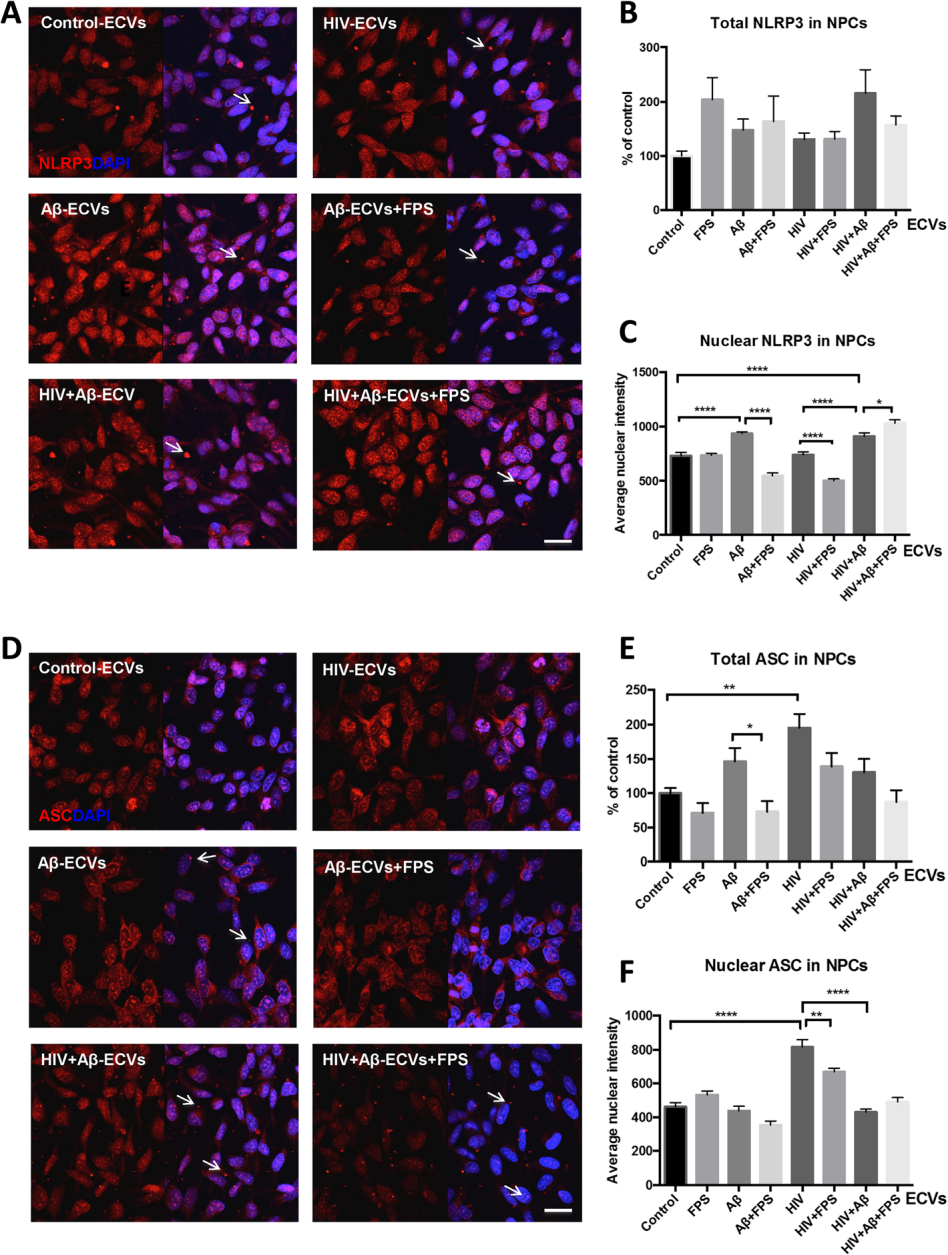
ECV-mediated Aβ transfer is associated with inflammasome protein changes in NPC. HBMEC were treated, and ECVs were isolated as in Fig. 2, followed by exposure to NPCs for 24 h. **a** Representative images of NLRP3 immunoreactivity (red) in NPCs by confocal microscopy. Nuclei are stained with DAPI (blue). Scale bar: 20 μm. **b** Quantification of total and **c** nuclear NLRP3 from the confocal images. **d** Representative images of ASC immunoreactivity (red) in NPCs by confocal microscopy. Nuclei are stained with DAPI (blue). Scale bar: 20 μm. **e** Quantification of total and **f** nuclear ASC from the confocal images. Values are mean ± SEM, n = 3–7 (total); *n* = 47–86 (nuclear). Statistically significant at *p < 0.05, **p < 0.01, ***p < 0.001, or ****p < 0.0001

Immunofluorescence microscopy for NLRP3 and ASC in NPCs after 24 h ECV treatment revealed unexpected and unusual nuclear localization of both proteins (Fig. 3). NLRP3 immunoreactivity was mostly nuclear with a finer granular pattern (Fig. 3a). In addition, a small number of brighter cytoplasmic or extracellular immunoreactivity dots were apparent in all experimental groups (Fig. 3a, arrows). While the total NLRP3 expression was not affected by the employed treatment (Fig. 3b and Additional file 1: Figure S3), NLRP3 levels significantly increased in the nuclei of NPCs as the result of Aβ-ECV and HIV + Aβ-ECV exposure (Fig. 3c). RAGE inhibition significantly decreased nuclear NLRP3 expression in NPCs exposed to Aβ-ECVs but modestly increased NLRP3 levels following HIV + Aβ-ECV treatment (Fig. 3c).

Regarding ASC expression, its immunoreactivity was distributed overwhelmingly in the nuclear area with an intense granular pattern consisting of different sized nuclear granules in control cells. Following Aβ-ECV exposure, this pattern remained primarily nuclear; however, several bright punctate immunoreactivity spots localized to the cytoplasm or the extracellular space appeared (Fig. 3d, arrows). In NPCs treated with HIV + Aβ-ECVs, alike ASC immunoreactive specks of different sizes were observed (arrows) in a similar location. Interestingly, the ASC specks were also visualized in the nuclei of cells exposed to HIV + Aβ-ECVs+FPS along with comparable immunoreactive patterns in the cytoplasm and/or extracellular space (arrows).

Total ASC immunoreactivity significantly increased in NPCs treated with HIV-ECVs. Interestingly, RAGE inhibition significantly decreased ASC levels after Aβ-ECVs exposure (Fig. 3e). Nuclear ASC expression largely mirrored the changes in the total levels of this protein, as HIV-ECVs induced the highest increase in ASC immunoreactivity compared to controls. RAGE inhibition decreased nuclear ASC in NPCs exposed to HIV-ECVs in the presence of FPS as compared to treatment with HIV-ECVs alone (Fig. 3f).

### RAGE modulates nuclear colocalization of Aβ with inflammasome proteins

**Fig. 4:**
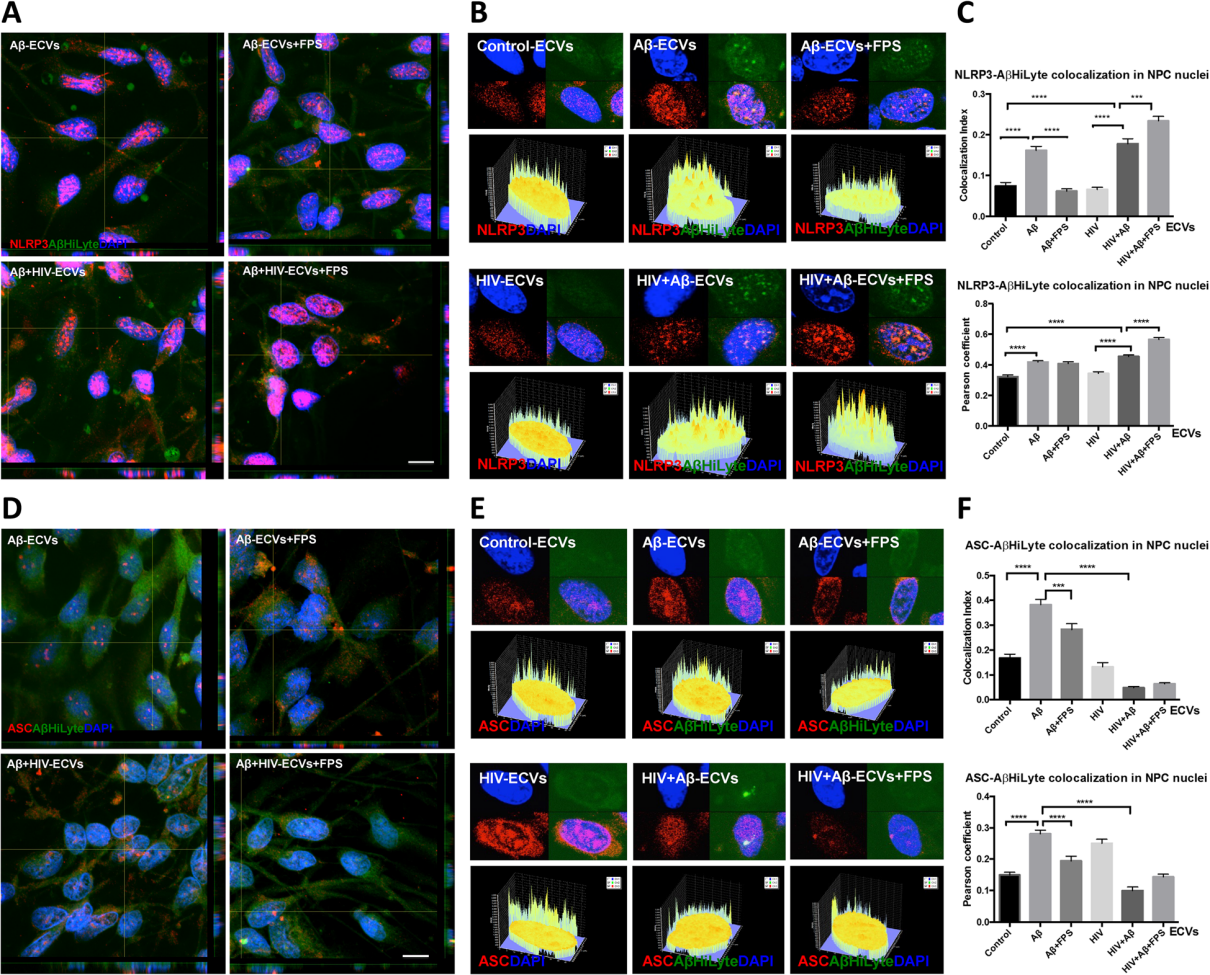
Aβ colocalization with inflammasome proteins in ECV-exposed NPCs. HBMEC were treated, and ECVs were isolated as in Figs. 2 and 3, followed by exposure to NPCs for 24 h. **a** NLRP3 immunoreactivity (red) and transferred Aβ (1–40) HiLyte (green) in NPCs as visualized by confocal microscopy. Nuclei are stained with DAPI (blue). Scale bar: 10 μm. **b** Nuclear colocalization of NLRP3 with Aβ (1–40) HiLyte. The graphs below the representative nuclear images depict fluorescence intensity profiles for colocalization of NLRP3 and Aβ (1–40) HiLyte in nuclear areas. **c** Quantification of NLRP3 and Aβ (1–40) HiLyte colocalization from B. **d** ASC immunoreactivity (red) and transferred Aβ (1–40) HiLyte (green) in NPCs as visualized by confocal microscopy. Nuclei are stained with DAPI (blue). Scale bar: 10 μm. **e** Nuclear colocalization of ASC with Aβ (1–40) HiLyte. The graphs below the representative nuclear images depict fluorescence intensity profiles for colocalization of ASC and Aβ (1–40) HiLyte in nuclear areas. **f** Quantification of ASC and Aβ (1–40) HiLyte colocalization from E. **a** & **d** Representative images from three experiments. **c**, **f** Values are mean ± SEM, *n* = 45–60. Statistically significant as compared to control at ***p < 0.001 or ****p < 0.0001

Knowing that exposure to HIV and/or Aβ -ECVs can alter distribution of inflammasome proteins in the nuclei of NPCs, we next evaluated, using the single cell resolution of confocal microscopy, whether Aβ colocalizes with nuclear NLRP3 or ASC upon the employed treatment factors. NPCs were treated as in Figs. 2 and 3 with ECVs isolated from Aβ and/or HIV-treated HBMEC as in Figs. 1, 2 and 3. Figure 4a depicts representative Z-stack images of colocalization of NLRP3 (red fluorescence), Aβ HiLyte (green fluorescence), and nuclei (DAPI, blue fluorescence). The majority of this colocalization occurs in the nuclei, and Fig. 4b illustrates the approach to quantify the nuclear Aβ/NLRP3 colocalization index and Pearson’s correlation coefficient, both shown on Fig. 4c. Exposure to Aβ-ECVs and HIV + Aβ-ECVs significantly increased NLRP3 colocalization with Aβ (Fig. 4c). The effect of RAGE inhibition was different in the absence or presence of HIV. Specifically, FPS blocked the colocalization of Aβ with NLRP3 when no HIV was present, i.e., in the Aβ-ECVs+FPS group as compared to the Aβ-ECV group, when analyzing the results by the colocalization index. In contrast, colocalization between Aβ and NLRP3 was significantly increased in the cells exposed to HIV + Aβ-ECVs+FPS as compared to HIV + Aβ-ECVs (Fig. 4c).

Figure 4d, e depict patterns of Aβ nuclear colocalization with ASC in NPCs treated with ECVs and/or FPS as in Fig. 4a-c. The colocalization was significantly increased as the result of Aβ-ECV exposure as compared to the background levels of control-ECVs (Fig. 4f). In addition, RAGE inhibition significantly blocked this effect. In the presence of ECVs isolated from HIV and Aβ-treated HBMEC, no such colocalization changes took place for ASC. The individual channels representing staining for NLRP3/ASC and Aβ HiLyte fluorescence from Fig. 4a and d are shown as Additional file 1: Figure S4. Overall, the results from Fig. 4 indicate that blocking RAGE activity in NPCs can profoundly impact Aβ-ECV and/or HIV-ECV-induced alterations of both ASC and NLRP3 localization to the nuclei of the recipient cells. In addition, ASC changes are consistent with the notion that in order to engage in the inflammasome pathway, a transfer from the nuclei into the cytoplasm is required [41]. Indeed, no changes in the inflammasome pathway end-players, such as caspase 1 activity and/or IL-1β levels, were detected after the employed ECV exposure (Additional file 1: Figure S3C-E), suggesting that the classical inflammasome pathway was not induced in NPCs.

### RAGE inhibition selectively affects differentiation of NPCs to mature neurons

In the next series of experiments, we evaluated the impact of the employed treatment factors on differentiation of NPCs to mature neurons. The rationale of these experiments was enhanced by a report that Aβ can affect neuronal differentiation via the inflammasome pathway involving RAGE [25]. NPCs were differentiated for 3 days as previously described [42, 43] in the presence of ECVs and/or FPS as in Figs. 2, 3 and 4. Cell differentiation was evaluated by counting cells positive for the neuronal marker Hu C/D [44], neuronal nuclear antigen (NeuN), a marker for mature neurons [45], and doublecortin (DCX), a marker of immature neurons [46]. In addition, the nuclei were stained with DAPI (blue). At least 9 images for every experimental condition from different samples were randomly acquired.

**Fig. 5:**
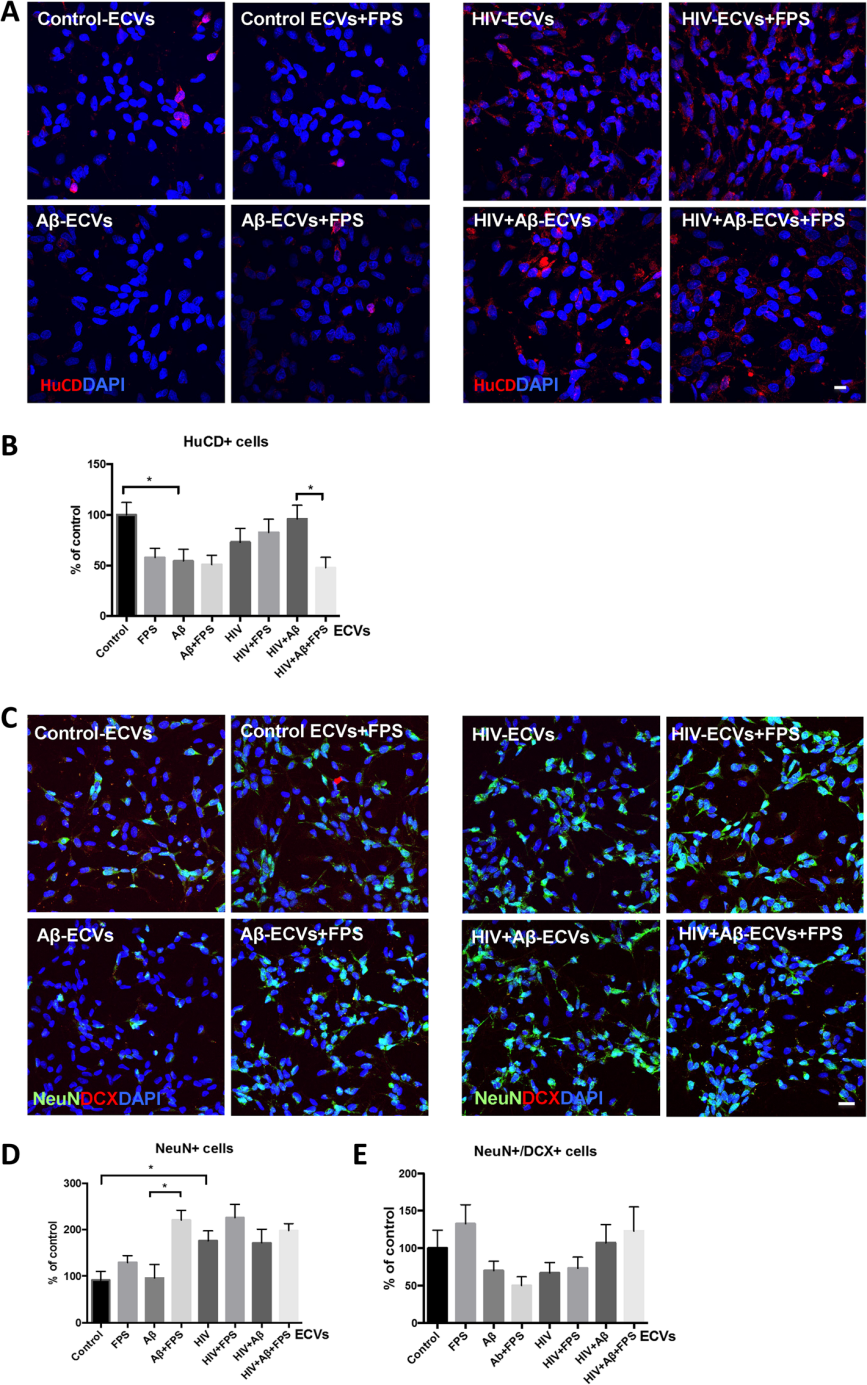
Impact of ECVs on NPC differentiation. HBMEC were treated with HIV and/or Aβ, and ECVs were isolated as in Fig. 1. NPC were differentiated for 3 days in the presence of HBMEC-derived ECVs. At the beginning of differentiation, selected NPC cultures were pretreated with 500 nM FPS-ZM1 (FPS) for 2 h followed by cotreatment with the isolated ECVs. At the end of the 3-day differentiation, confocal microscopy was performed for neuronal markers. Neuronal differentiation was assessed by counting Hu C/D-, NeuN- and doublecortin (DCX) positive cells. At least 9 images for every experimental condition from different samples were randomly acquired. Scale bar: 20 μm. **a** Representative images of Hu C/D immunoreactivity (red); nuclei are stained with DAPI (blue). **b** Hu C/D positive cells were counted from confocal microscopy images. **c** Representative images of NeuN (green) and doublecortin (DCX, red) immunoreactivity; nuclei are stained with DAPI (blue). **d** NeuN positive and **e** NeuN/DCX double positive cells were counted from confocal microscopy images. Values are mean ± SEM, *n* = 30–43 (Hu C/D); *n* = 7–15 (NeuN); *n* = 13 (NeuN/DCX). *Statistically significant as compared to control at *p* < 0.05

A prominent nuclear Hu C/D staining pattern was detected in the differentiated control cells; however, the number of Hu C/D-positive cells was markedly diminished by exposure to ECVs derived from Aβ-treated HBMEC, suggesting impaired neuronal maturation (Fig. 5a, b). Interestingly, the number of Hu C/D-positive cells was also significantly lower in the HIV + Aβ-ECVs+FPS group when compared to the HIV + Aβ-ECVs group, indicating that RAGE inhibition in the recipient NPCs diminished neuronal differentiation in response to Aβ-ECV transfer only in the presence of HIV (Fig. 5b). In addition, Additional file 1: Figure S5A-B reflect quantified total and nuclear intensity of Hu C/D immunoreactivity in differentiated NPCs. Due to high standard deviation, there was no significant nuclear colocalization change between Hu C/D and Aβ in any of the studied groups (Additional file 1: Figure S5C).

Representative colocalization images of NeuN (green) and DCX (red) are presented on Fig. 5c. Quantification of the DCX- and/or NeuN-positive cells is illustrated in Fig. 5d-e, respectively. The number of NeuN positive cells was not affected by the Aβ-ECVs treatment; however, it was significantly increased in the presence of HIV-ECVs as compared to the control. RAGE inhibition significantly increased NeuN positive cell number in the Aβ-ECVs+FPS group as compared to the Aβ-ECVs group (Fig. 5d). The number of NeuN/DCX double positive cells did not change significantly as the result of the employed treatment factors (Fig. 5e). Overall, these results suggest that Aβ-ECVs, HIV-ECVs, and/or RAGE inhibition have a modulatory effect on neuronal differentiation. The impact of RAGE inhibition on NPC differentiation fate is complex. On one hand, decreasing Hu C/D immunopositive cell number after Aβ-ECVs transfer in the presence of HIV; on the other hand, increasing the number of NeuN positive cells after Aβ-ECV treatment without HIV.

### Aβ- or HIV-ECVs-induced alterations of NPC differentiation are not associated with changes in soluble proinflammatory mediators

**Fig. 6:**
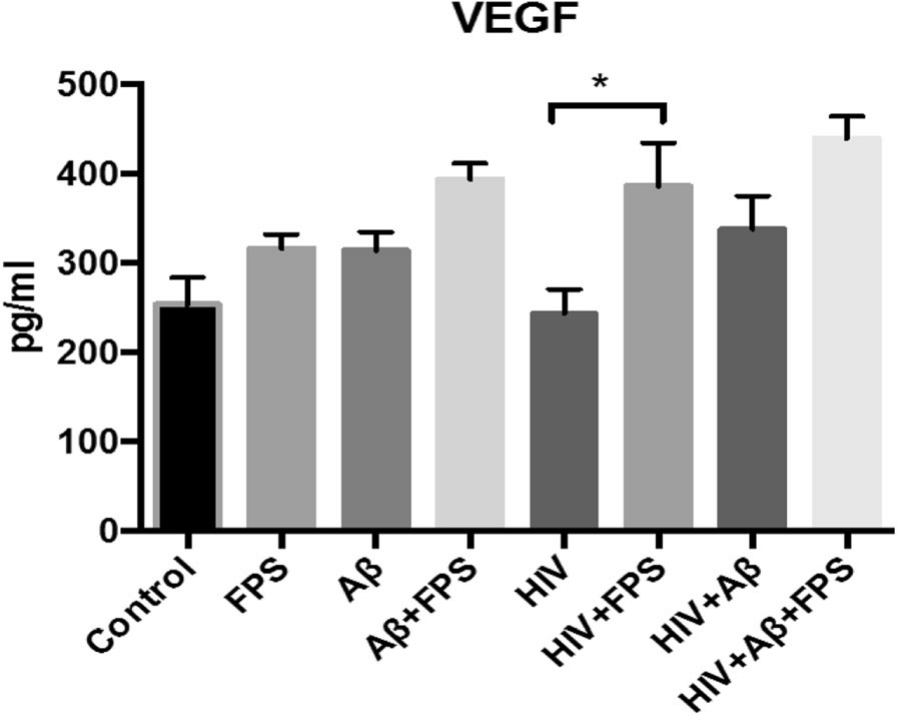
Cytokine/chemokine panel after NPC differentiation. NPC were treated and differentiated as in Fig. 5. At the end of the 3-day differentiation, a cytokine/chemokine panel (G-CSF, IL-4, MCP-1, Fractalkine, PDGF-AA, PDGF-AB/BB, VEGF-A, BDNF, NGF-β) was measured by Luminex MagPix assay or ELISA from the cell culture media. Among the detected factors, only VEGF-A levels were significantly altered. Values are mean ± SEM, *n* = 5–8. *Statistically significant at *p* < 0.05

In the final series of experiments, we measured a panel of cytokines/chemokines/growth factors from the conditioned culture media of exposed NPCs after 3 days of differentiation. The panel included Fractalkine, G-CSF, IL-4, CCL-2, PDGF-AA, PDGF-AB/BB, and VEGF-A. We choose this panel based on a previous report suggesting its susceptibility to changes due to the presence or absence of bFGF, i.e., one of the growth factors that are being withdrawn from the culture medium to initiate differentiation [47]. Among the studied active compounds, the only significant change was an increase in VEGF-A levels in the media of differentiated NPCs exposed to HIV-ECVs+FPS as compared to HIV-ECVs (Fig. 6 and Additional file 1: Figure S6).

**Fig. 7:**
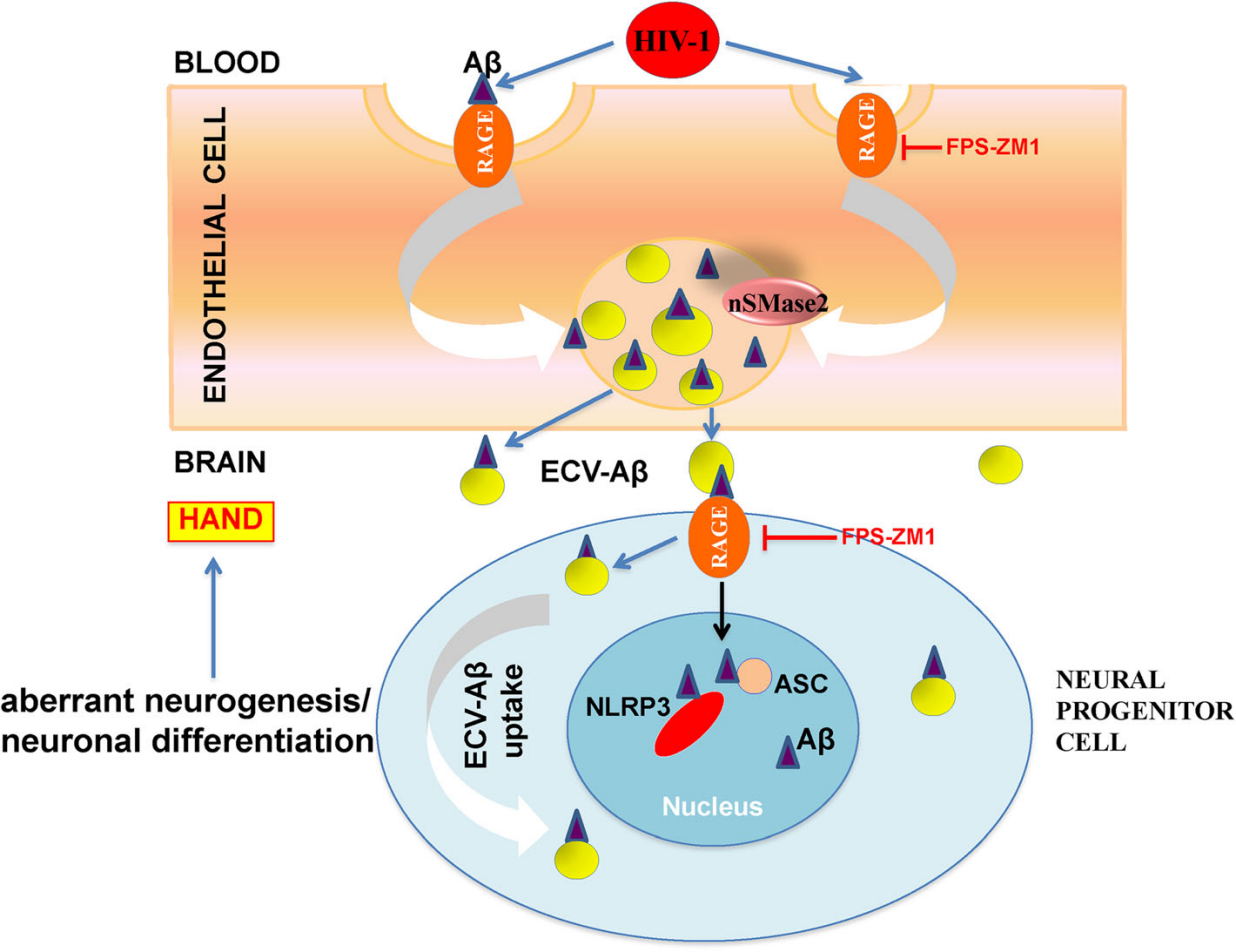
Schematic diagram of the involvement of RAGE in brain endothelial ECV release and ECV-mediated Aβ transfer to NPC. Our data indicate that RAGE affects HIV-induced brain endothelial ECV release and ECV-mediated Aβ transfer to NPC. Aβ uptake by NPC nuclei and colocalization with the inflammasome markers (NLRP3, ASC) are modulated by RAGE. These complex events affect NPC differentiation into neurons and may contribute to HIV associated neurocognitive disorders. Abbreviations: Aβ, amyloid beta; ECV, extracellular vesicle; HAND, HIV associated neurocognitive disorders; NPC, neural progenitor cell; RAGE, receptor for advanced glycation end products

We also measured the brain-derived neurotrophic factor (BDNF) and the nerve growth factor-β (NGF-β) because they provide signaling and trophic support during NPC differentiation [48]. The levels of these growth factors were not detectable in NPC culture media 3 days after differentiation.

## Discussion

ECVs are recognized as the carriers of biologically active proteins and genetic materials, such as mRNA, microRNA, siRNA, and DNA, which implicate them in the physiology and pathology of the CNS. However, the mechanisms of ECVs generation by the parent cells and the mechanisms of their uptake by the recipient cells are not fully understood. In this work, we examined two critical aspects of the involvement of RAGE in the ECV-mediated Aβ pathology in the context of HIV infection. First, we evaluated the role of RAGE in endothelial cell-derived ECV release and Aβ-ECV levels. Second, we examined the involvement of RAGE in the ECV-mediated transfer of Aβ to the neighboring neuronal progenitor cells (NPC) and its effects on neuronal differentiation.

Novel findings of the present study indicated that inhibition of RAGE in brain endothelial cells resulted in a significant increase in produced ECVs (Fig. 1a, b), and a decrease in Aβ and protein levels in ECVs (Fig. 1d-f). These changes appeared to be specific because we did not observe any alterations in ECV number upon nSMase inhibition. Our results on the apparent lack of the role of nSMase in ECV production by HBMEC are in contrast to the literature reports that implicated this pathway in exosome generation [38] and in secretion of microvesicles from macrophages [27]. On the other hand, inhibition of nSMase decreased Aβ-ECV levels, mimicking the input of RAGE inhibition, and suggesting that these pathways may be related in regulation of cellular Aβ release via ECVs.

ECVs derived from brain endothelial cells can transfer Aβ to other cells of the neurovascular unit, such as astrocytes and pericytes [22]. Therefore, we hypothesized that ECVs may also deliver Aβ to other neighboring cells, such as NPCs found in neurogenic niches in the perivascular space and close to the brain microvasculature [24]. Indeed, novel results reported in the current study indicate that ECVs can transfer Aβ from the brain endothelial cells to NPCs, and that this process is facilitated in the context of HIV (Fig. 2). Aβ was also delivered to the nuclei of the recipient NPCs, a process that can result in profound transcriptomic changes [49]. Furthermore, knowing that NPCs express RAGE [25], we evaluated the role of RAGE in Aβ transfer to NPCs. While the total Aβ transfer was not statistically affected by blocking RAGE, inhibition of this receptor significantly reduced Aβ transfer to the nuclear compartment. Interestingly, a regulatory role of RAGE in Aβ transfer was apparent only in the context of HIV (Fig. 2c), an observation that is consistent with the role of this transfer system in HIV infection [50].

In the next series of experiments, we investigated the implications of Aβ transfer to NPCs by evaluating the inflammasome pathway and differentiation of NPCs to mature neurons. The inflammasomes are cytoplasmic complexes containing the danger signal sensing proteins of the NLR family, such as NLRP3 evaluated in the present study. The NLR proteins can then recruit the adaptor ASC (apoptosis-associated speck-like protein containing a caspase recruitment domain) and form cytoplasmic complexes, leading to activation of caspase-1 as well as release of IL-1β and IL-18 [51].

Exposure to HIV-ECVs significantly increased the total and nuclear ASC levels. In addition, NLRP3 nuclear levels increased significantly after HIV + Aβ-ECVs exposure as compared to control. These results are consistent with the reported role of inflammasome in HIV infection [28, 52–54]. For example, NLRP3 and IL-1β polymorphisms were linked to an increased susceptibility to HIV infection [55, 56]. ASC specs were found in the plasma of HIV positive patients [57], and infection with HIV stimulated the NLRP3 inflammasome in monocytes [58] as well as production of caspase-1, IL-1β and IL-18 in brain microglial cells [59]. HIV viral proteins, such as Vpr and Tat, were also demonstrated to activate the NLRP3 inflammasome in human microglia [40, 60]. Finally, activation of the NLRP3 inflammasome in human monocytes has been shown in response to antiretroviral treatment [61].

Our novel observations also indicate that exposure to Aβ-ECVs and HIV + Aβ-ECVs increased nuclear localization of NLRP3. This effect was associated with elevated Aβ levels as the result of ECV-mediated Aβ transfer to the recipient NPCs and was consistent with the role of the NLR proteins to recognize misfolded proteins, such as Aβ. Indeed, Aβ was shown to activate the inflammasome pathway in LPS-primed macrophages [29]. In addition, when NLRP3-deficient mice were crossed with APP/PS1 transgenic mice, the offspring had no amyloid plaques and their neurobehavioral performance improved, suggesting a role of the NLRP3 inflammasome in the Aβ pathology [62]. This notion was further supported by findings that inflammasome markers colocalized with Aβ in human AD brains [30], and ASC was present in the core of amyloid plaques in both the mouse and human brains [63]. Moreover, ASC specs from microglia can cross-seed Aβ and facilitate the formation of Aβ oligomers and aggregates in vitro. Injection of ASC specs in the hippocampus of Alzheimer’s disease mice initiates spreading of Aβ pathology in the brain. This close connection between Aβ and the inflammasome is further supported by the observations that Aβ colocalized with both ASC and NLRP3 in the nuclei of NPCs, the effect that was modulated by RAGE inhibition.

While NPCs strongly expressed inflammasome proteins such as NLRP3 and ASC, the expression of these proteins was mostly localized to nuclei, with occasional brighter ASC specks in the cytosol or in the extracellular space in the Aβ-ECVs, HIV + Aβ-ECVs and HIV + Aβ-ECVs+FPS groups. The nuclear localization of NLRP3 and ASC cannot fully explain their potential role in the inflammasome assembly, which takes place in the cytosol. In fact, it was proposed that inducible redistribution of ASC from the nucleus to the cytoplasm is necessary for the inflammasome response [41, 64]. These observations are in line with the fact that no caspase-1 activation was observed in NPCs after the employed treatment, and the levels of IL-1β were inconsistent; however, mostly negative under these conditions. RAGE inhibition modulated levels and cellular localization of both ASC and NLRP3 in response to Aβ and/or HIV-ECVs exposure, supporting the notion that RAGE can signal through the NLRP3 inflammasome [31].

Although both Aβ and HIV pathology was linked to the NLRP3 inflammasome, i.e., the pathway that was examined in the present study, other types of inflammasomes may also be involved in the observed effects. For instance, AIM2 is the dominant inflammasome sensor in the mouse brain. Its deletion caused a decrease in Aβ deposition and microglial activation along with IL-6 and IL-18 increase [65]. In the serum of HIV patients with higher viral load, AIM2 gene expression increased along with NLRP3, ASC, IL-1β, and IL-18 [66]. In another report Aβ oligomers were demonstrated to disturb the neuronal membrane causing K+ efflux from the cell. The low intracellular K+ concentration may activate the NALP1 inflammasome leading to IL-1β and IL-18 increase [67]. In fact, Aβ was shown to induce NLRP1-dependent neuronal pyroptosis, as NLRP1 was upregulated in cultured cortical neurons leading to caspase-1 dependent pyroptosis [68]. Recently, HIV Tat was shown to downregulate NLRC5 in vitro via the miRNA-34a-NLRC5-NFκB signaling axis leading to an IL-1β level increase. NLRC5 was also downregulated in HIV transgenic rats and SIV infected macaques [69].

Another long-term outcome of Aβ transfer to NPCs may be aberrant differentiation of these cells to mature neurons. The process is important as adult hippocampal neurogenesis was demonstrated to play a role in learning and memory [70, 71] and neurocognitive dysfunction was linked to aberrant NPC neurogenesis [72]. Novel findings of the current study indicate that both Aβ-ECVs and RAGE inhibition altered NPC differentiation. Specifically, the number of HuC/D positive cells was significantly decreased in NPCs exposed to Aβ-ECVs. Interestingly, RAGE inhibition also decreased Hu C/D+ cell number in the HIV + Aβ-ECVs+FPS group as compared to the HIV + Aβ-ECVs group. On the other hand, RAGE inhibition increased the number of NeuN positive cells in the Aβ-ECVs+FPS group versus the Aβ-ECV group. These results indicate the complex effects of Aβ-ECVs on NPC differentiation with and without HIV; however, they are consistent with the observations that Aβ profoundly affects NPC differentiation and the inflammasome via the Aβ-(HMGB-1)-RAGE/NF-κB-NLRP3 pathway, further confirming the findings from the current study. The mechanisms of this effect are related to the fact that NF-κB can bind to the NLRP3 promoter followed by inflammasome activation, neuroinflammation, and neurotoxicity [25]. In our experiments, exposure to HIV-ECVs increased NeuN positive cell number. These results are in line with the literature reports demonstrating that NPCs could be infected with HIV [73–75] and HIV affected their proliferation and survival in vitro and in vivo [73, 76]. Moreover, the number of hippocampal NPCs in postmortem brains in HIV patients with dementia was lower when compared to patients without dementia [26].

In summary, results of the present study indicate that ECVs can efficiently transfer Aβ from brain endothelial cells to the neighboring NPCs. Importantly, this process is influenced by HIV and regulated, at least in part, by RAGE (Fig. 7). The long-term effect of Aβ transfer to NPCs includes alterations of NPC differentiation via a mechanism that may involve changes to the inflammasome machinery. Overall, these changes may contribute to the development of neurocognitive impairment and Aβ pathology in HIV-infected brains.

## Supplementary information

**Additional file 1: Figure S1.** HBMEC-derived ECVs. **Figure S2.** The effects of nSMase inhibition on brain endothelial ECV release and ECV-Aβ levels. **Figure S3.** Analysis of NLRP3, ASC, caspase-1, and IL-1β. **Figure S4.** Impact of HBMEC-derived ECVs on colocalization of NLRP3 and ASC with Aβ in NPCs. **Figure S5.** Impact of ECV-mediated Aβ transfer on NPC differentiation as measured by HuC/D. **Figure S6.** Cytokine/chemokine panel after NPC differentiation.

## Data Availability

The datasets analyzed during the current study available from the corresponding author upon request.

## Abbreviations

AD: Alzheimer’s disease
ASC: Apoptosis-associated speck-like protein containing CARD
Aβ: Amyloid beta
BBB: Blood-brain barrier
BDNF: Brain-derived neurotrophic factor
CCL2: C-C motif chemokine ligand 2
DAPI: 4′,6-diamidino-2-phenylindole
DCX: Doublecortin
ECVs: Extracellular vesicles
EGF: Epidermal growth factor
ELISA: Enzyme linked immunosorbent assay
FPS: FPS-ZM1
G-CSF: Granulocyte colony stimulating factor
HAND: HIV-associated neurocognitive disorders
HBMEC: Human brain microvascular endothelial cells
HIV: Human immunodeficiency virus type 1
HuCD: Human HuC/HuD neuronal protein
IL-18: Interleukin 18
IL-1β: Interleukin 1 beta
IL-4: Interleukin 4
LPS: Lipopolysaccharide
NeuN: Neuronal nuclear antigen
NGF-β: Nerve growth factor beta
NLRP3: NLR family pyrin domain containing 3
NPCs: Neural progenitor cells
nSMase: Neutral sphingomyelinase
NTA: Nanoparticle tracking analysis
PBS: Phosphate buffered saline
PDGF-AA: Platelet derived growth factor-AA
PDGF-AB/BB: Platelet derived growth factor-AB/BB
RAGE: Receptor for advanced glycation end products
VEGF-A: Vascular endothelial growth factor A
