# Supplementary material for: Extracellular vesicle-mediated amyloid transfer to neural progenitor cells: implications for RAGE and HIV infection
Source: Mol Brain. 2020 Feb 17;13:21. doi: 10.1186/s13041-020-0562-0 (PMC7027073; doi:10.1186/s13041-020-0562-0)
Supplement: Supplementary file 1 — Additional file 1: Figure S1. HBMEC-derived ECVs. Figure S2. The effects of nSMase inhibition on brain endothelial ECV release and ECV-Aβ levels. Figure S3. Analysis of NLRP3, ASC, caspase-1, and IL-1β. Figure S4. Impact of HBMEC-derived ECVs on colocalization of NLRP3 and ASC with Aβ in NPCs. Figure S5. Impact of ECV-mediated Aβ transfer on NPC differentiation as measured by HuC/D. Figure S6. Cytokine/chemokine panel after NPC differentiation. [file 13041_2020_562_MOESM1_ESM.pdf]

Extracellular vesicle-mediated amyloid transfer to neural progenitor cells: implications for RAGE and HIV infection

Ibolya E. András[1], Marta Garcia-Contreras[2] Christopher Yanick[1], Paola Perez[1], Brice Sewell[1], Leonardo Durand[1] and Michal Toborek[1]

[1]Department of Biochemistry and Molecular Biology, 1011 NW 15th Street; Gautier Building, Room 528; University of Miami School of Medicine, Miami, FL 33136-1019, USA, [2]Diabetes Research Institute, 1450 NW 10th Ave, University of Miami School of Medicine, Miami, FL 33136-1011, USA

Supplemental Figures

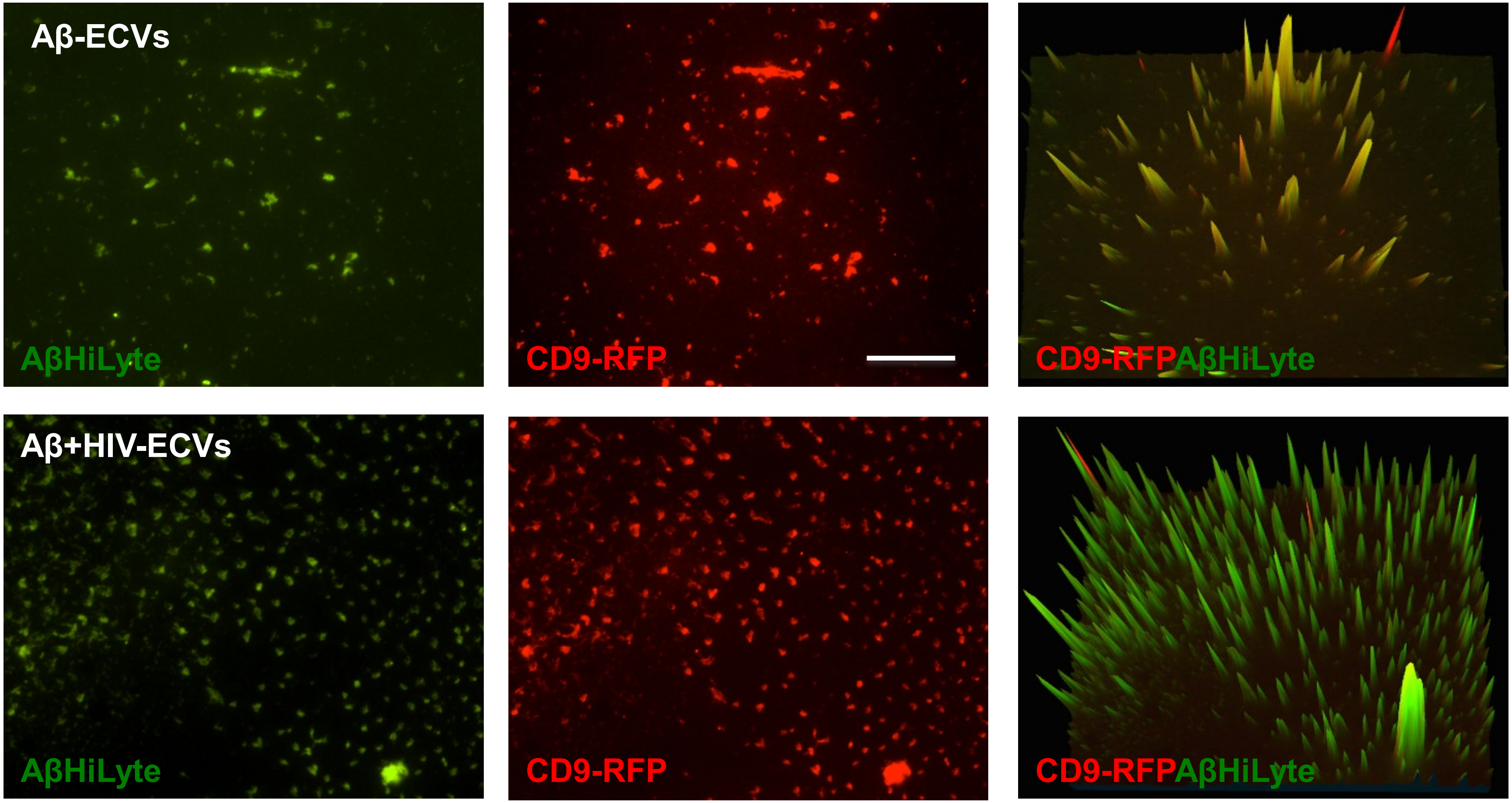

Figure S1. **HBMEC-derived ECVs.** HBMEC were transfected with the CD9 Cyto-Tracer construct pT CD9-RFP (System Biosciences) using Purefection Transfection Reagent following the manufacturer's protocol. Twenty four hours post transfection, cells were exposed to HIV (30 ng/ml) and/or 100 nM Aβ (1–40) HiLyte AlexaFluor-488 (green) for 48 h as described previously (Andras et al., Mol Cell Neurosci 79:12-22, 2017). Green and red fluorescent ECV were isolated from the culture media and imaged by fluorescence microscopy (scale bar: 20 µm). The right panels show the intensity surface plots for the merged images. Note that the green fluorescent Aβ is associated with ECVs of different sizes. Most of the CD9-RFP positive ECVs have fluorescent Aβ cargo.

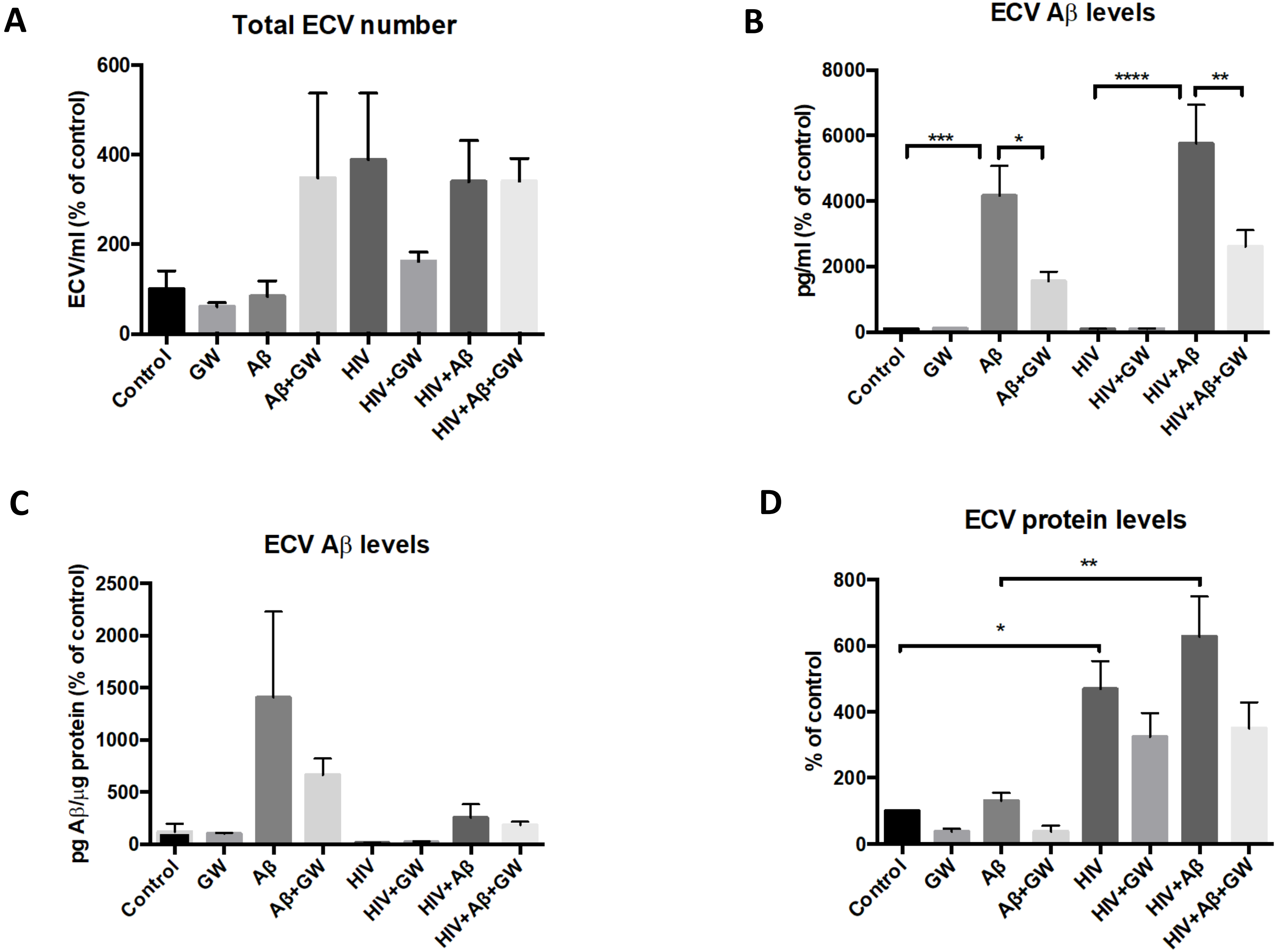

A
Total ECV number
ECV/ml (% of control)
0
200
400
600
Control
GW
Aβ
Aβ+GW
HIV
HIV+GW
HIV+Aβ
HIV+Aβ+GW
B
ECV Aβ levels
pg/ml (% of control)
0
2000
4000
6000
8000
***
*
****
**
C
ECV Aβ levels
pg Aβ/μg protein (% of control)
0
500
1000
1500
2000
2500
D
ECV protein levels
% of control
0
200
400
600
800
*
**

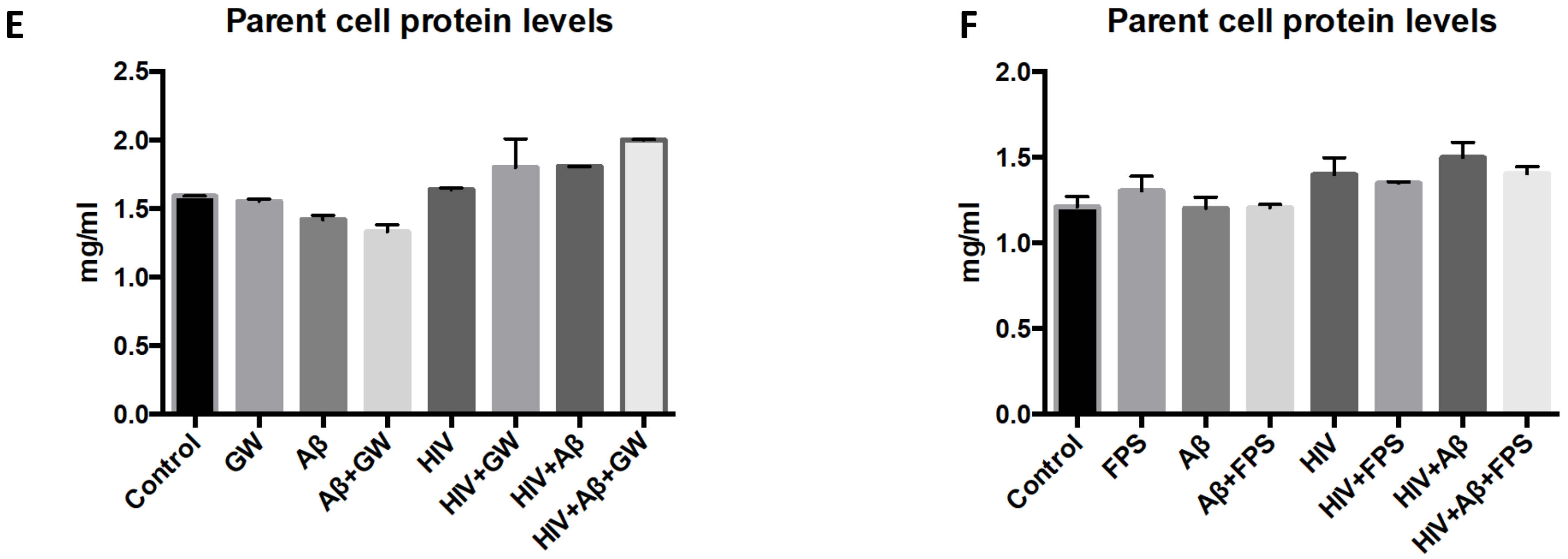

Figure S2. The effects of nSMase inhibition on brain endothelial ECV release and ECV-Aβ levels. HBMEC were exposed to 30 ng/ml HIV particles and/or 100 nM Aβ (1-40) for 48 h. Selected cultures were pretreated with 20 μM GW4869 for 1 h followed by cotreatment with 30 ng/ml HIV particles and/or 100 nM Aβ (1-40) for 48 h. ECVs were isolated from the culture media. **A)** Total ECV number as measured by nanoparticle tracking analysis (NTA). Values are mean ± SEM, n=3-4. **B & C)** Total ECV Aβ (1-40) levels as measured by ELISA and normalized either to **B)** media volume or **C)** ECV protein levels. Values are mean ± SEM, n=7. **D)** ECV protein levels as measured by BCA assay. **E&F)** Parent cell protein levels as measured by BCA assay. Values are mean ± SEM, n=3. Statistically significant at *p<0.05, **p<0.01, ***p<0.001, or ****p<0.0001.

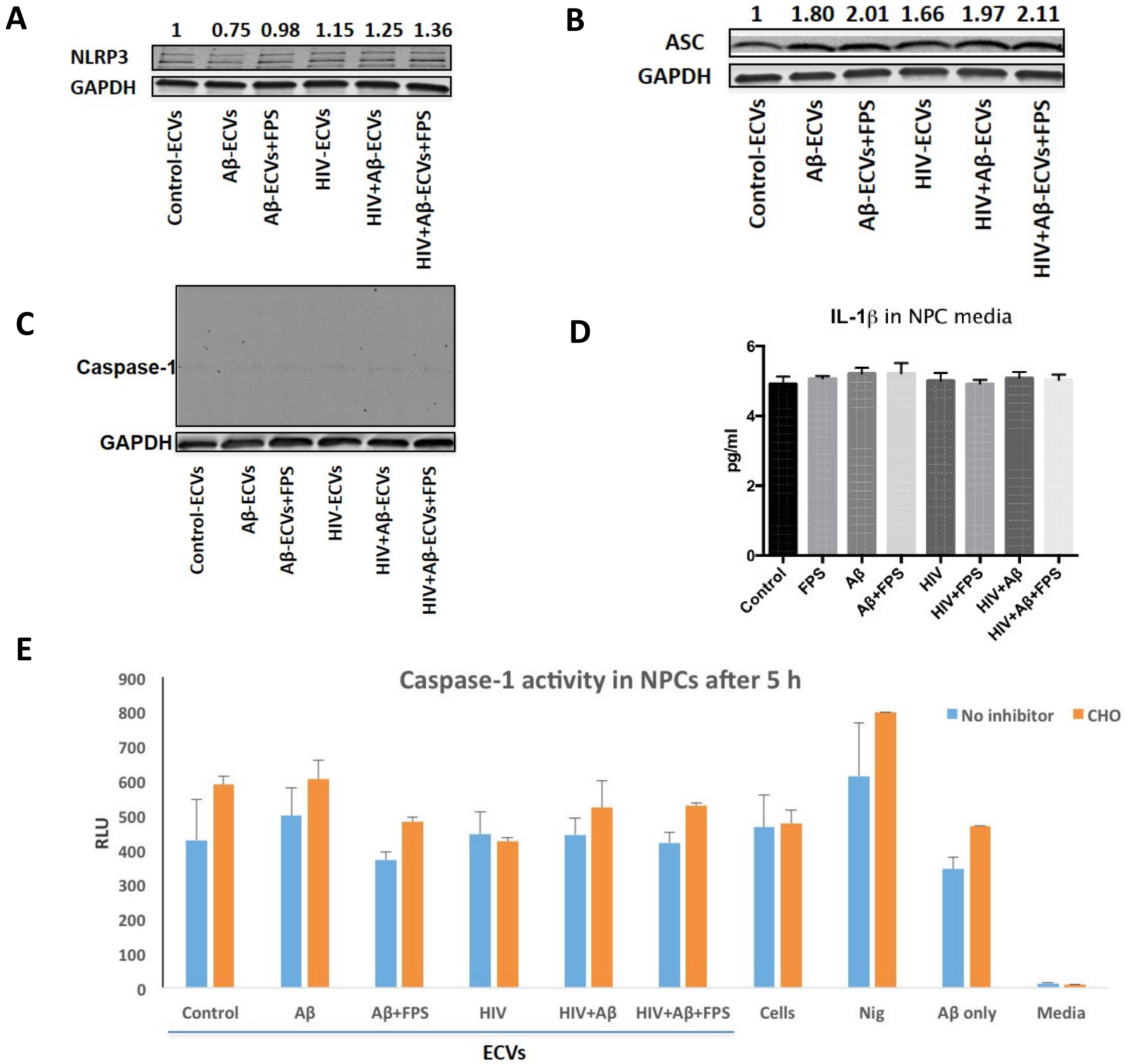

A
1 0.75 0.98 1.15 1.25 1.36
NLRP3
GAPDH
Control-ECVs
Aβ-ECVs
Aβ-ECVs+FPS
HIV-ECVs
HIV+Aβ-ECVs
HIV+Aβ-ECVs+FPS
B
1 1.80 2.01 1.66 1.97 2.11
ASC
GAPDH
Control-ECVs
Aβ-ECVs
Aβ-ECVs+FPS
HIV-ECVs
HIV+Aβ-ECVs
HIV+Aβ-ECVs+FPS
C
Caspase-1
GAPDH
Control-ECVs
Aβ-ECVs
Aβ-ECVs+FPS
HIV-ECVs
HIV+Aβ-ECVs
HIV+Aβ-ECVs+FPS
D
IL-1β in NPC media
pg/ml
0
2
4
6
Control
FPS
Aβ
Aβ+FPS
HIV
HIV+FPS
HIV+Aβ
HIV+Aβ+FPS
E
Caspase-1 activity in NPCs after 5 h
RLU
0
100
200
300
400
500
600
700
800
900
No inhibitor
CHO
Control
Aβ
Aβ+FPS
HIV
HIV+Aβ
HIV+Aβ+FPS
Cells
Nig
Aβ only
Media
ECVs

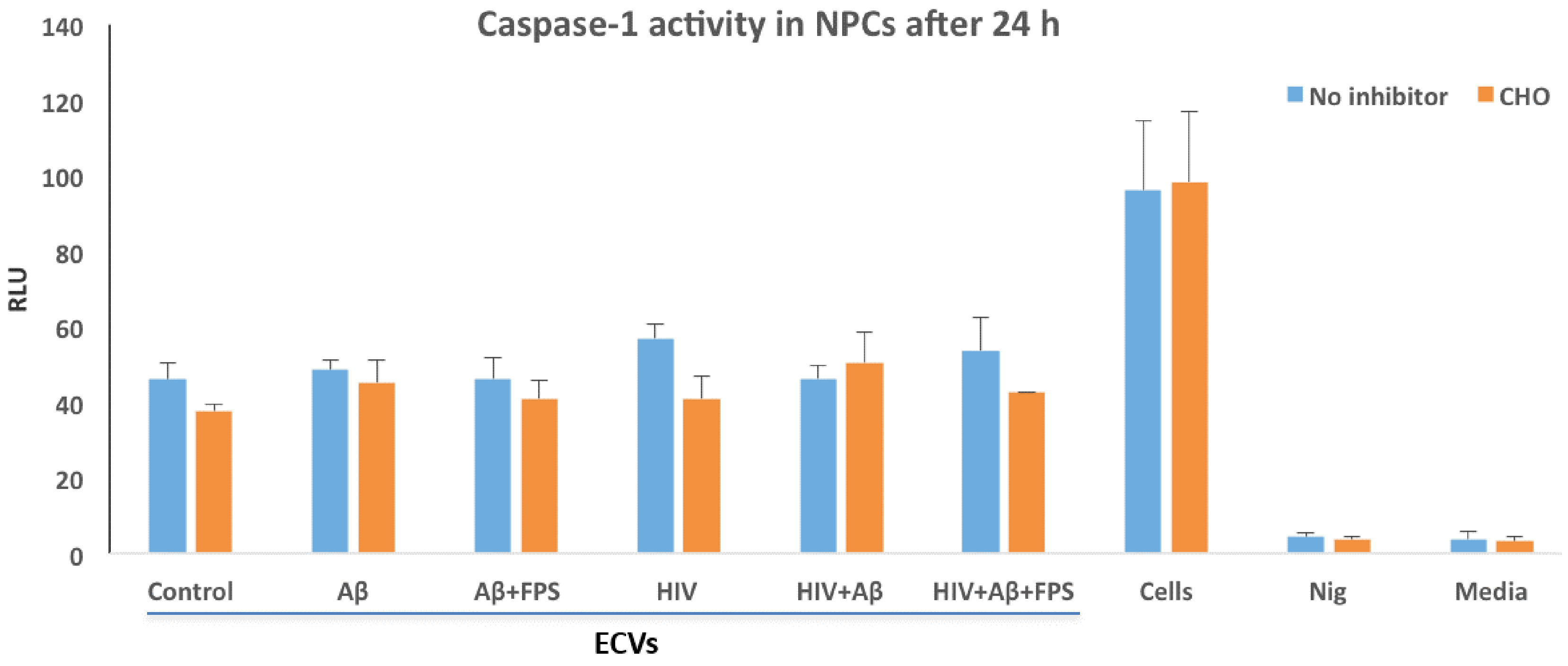

Figure S3. Analysis of NLRP3, ASC, caspase-1, and IL-1β. HBMEC were treated with HIV and/or Aβ and ECVs were isolated as in Figure S2. Then, human NPCs were exposed to HBMEC-derived NPCs for 24 h, with selected cultures pretreated with 500 nM FPS-ZM1 (FPS) for 2 h followed by cotreatment with the isolated ECVs. Immunoblotting for **A)** NLRP3, **B)** ASC and **C)** caspase-1. GAPDH was assessed as a loading control. **D)** IL-1β in the NPC culture media was undetectable 24 h after ECV exposure. IL-1β levels were detected once by ELISA 3 days after differentiation as shown on the graph. **E)** NPC caspase-1 activity was measured 5 h and 24 h after ECV exposure by the Caspase-Glo 1 inflammasome assay with or without the specific caspase-1 inhibitor YVAD-CHO (CHO). Cells without ECVs treatment were used as additional control. Nigericin (Nig, 20 µM) and Aβ (1 µM) were used as positive controls. Cell culture media alone was used as a negative control. Luminescence was recorded after 60 minutes. Values are mean ± SEM, n=4 (no inhibitor) and n=2 (CHO).

A

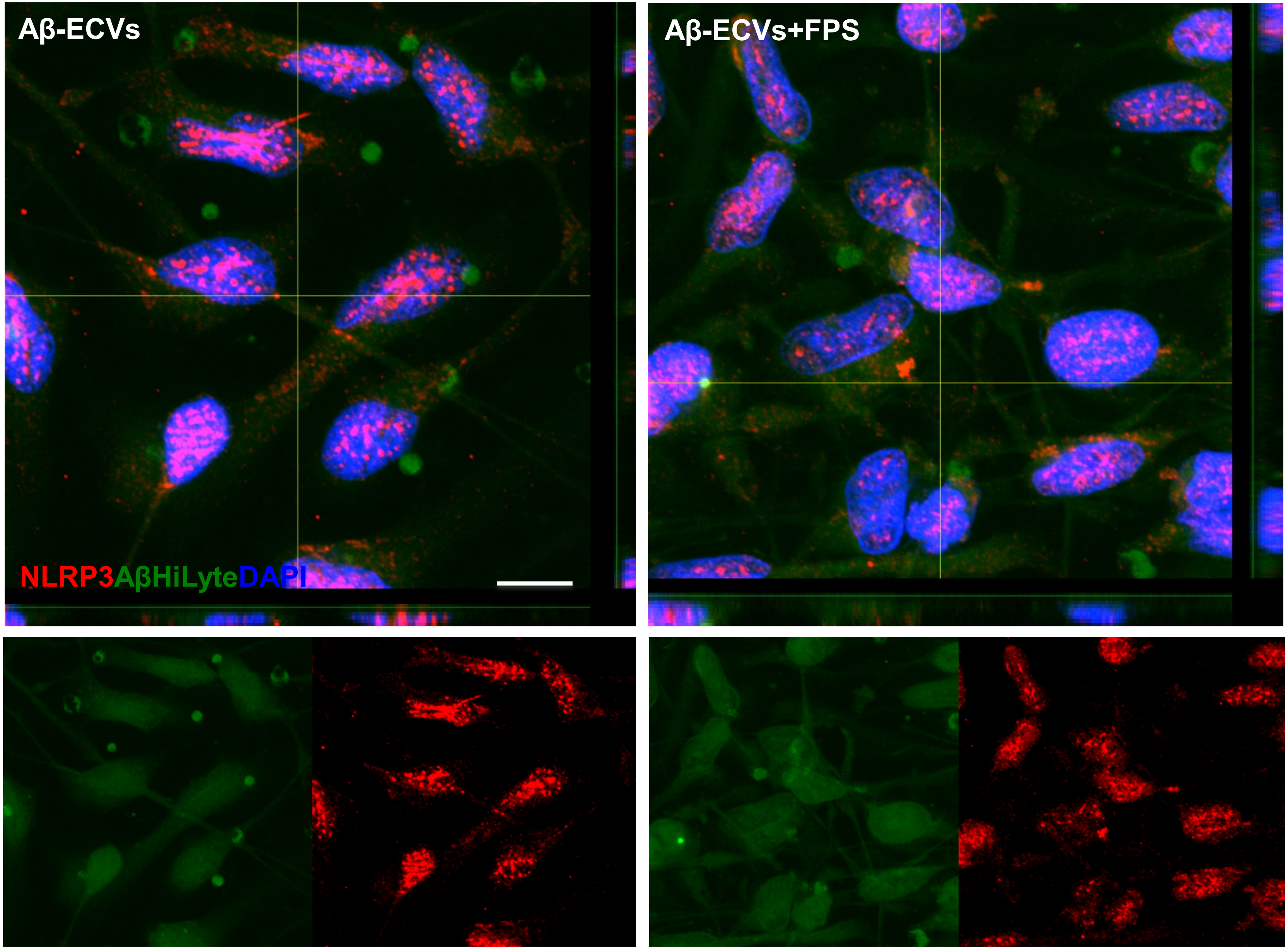

A

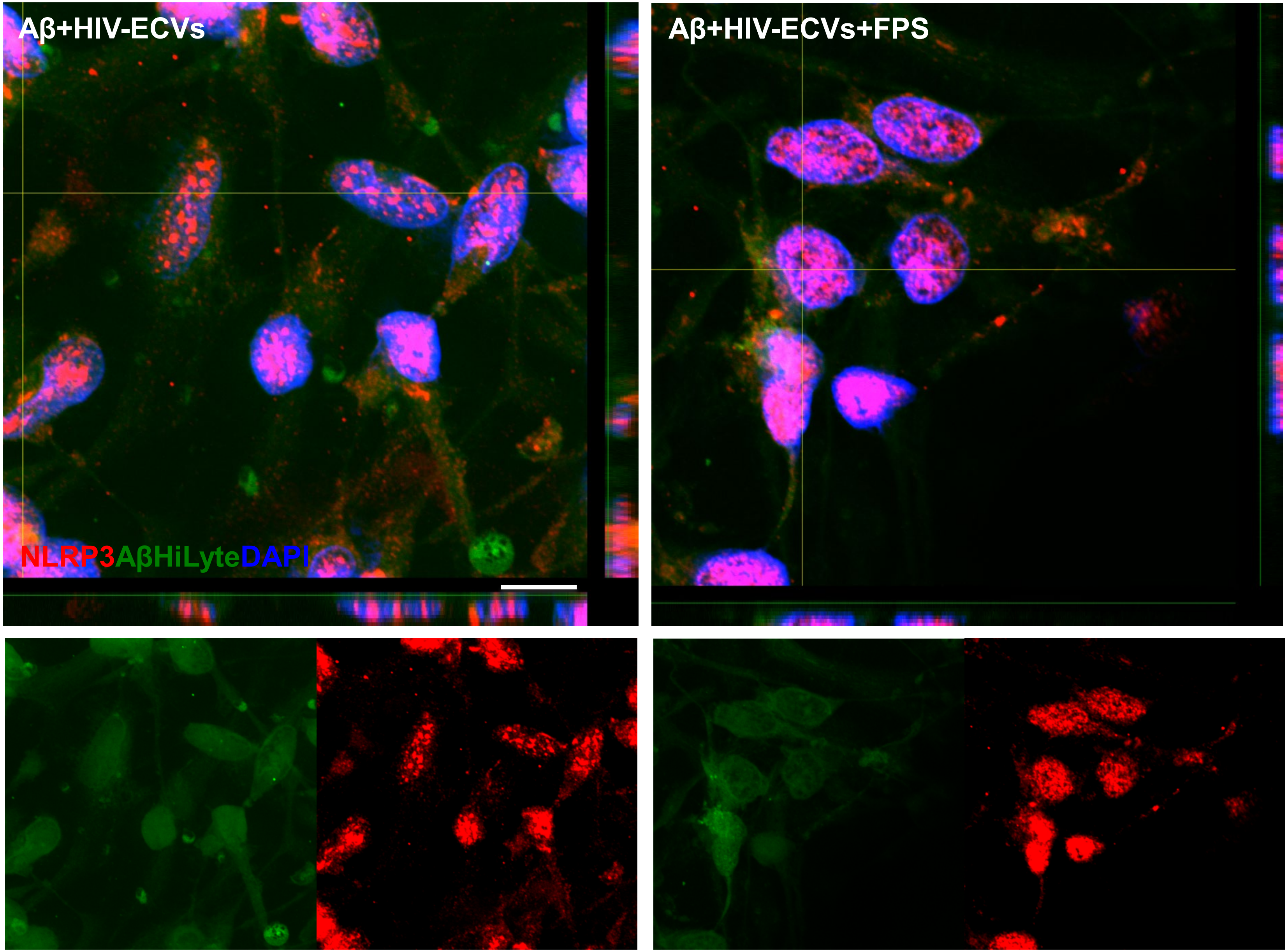

B

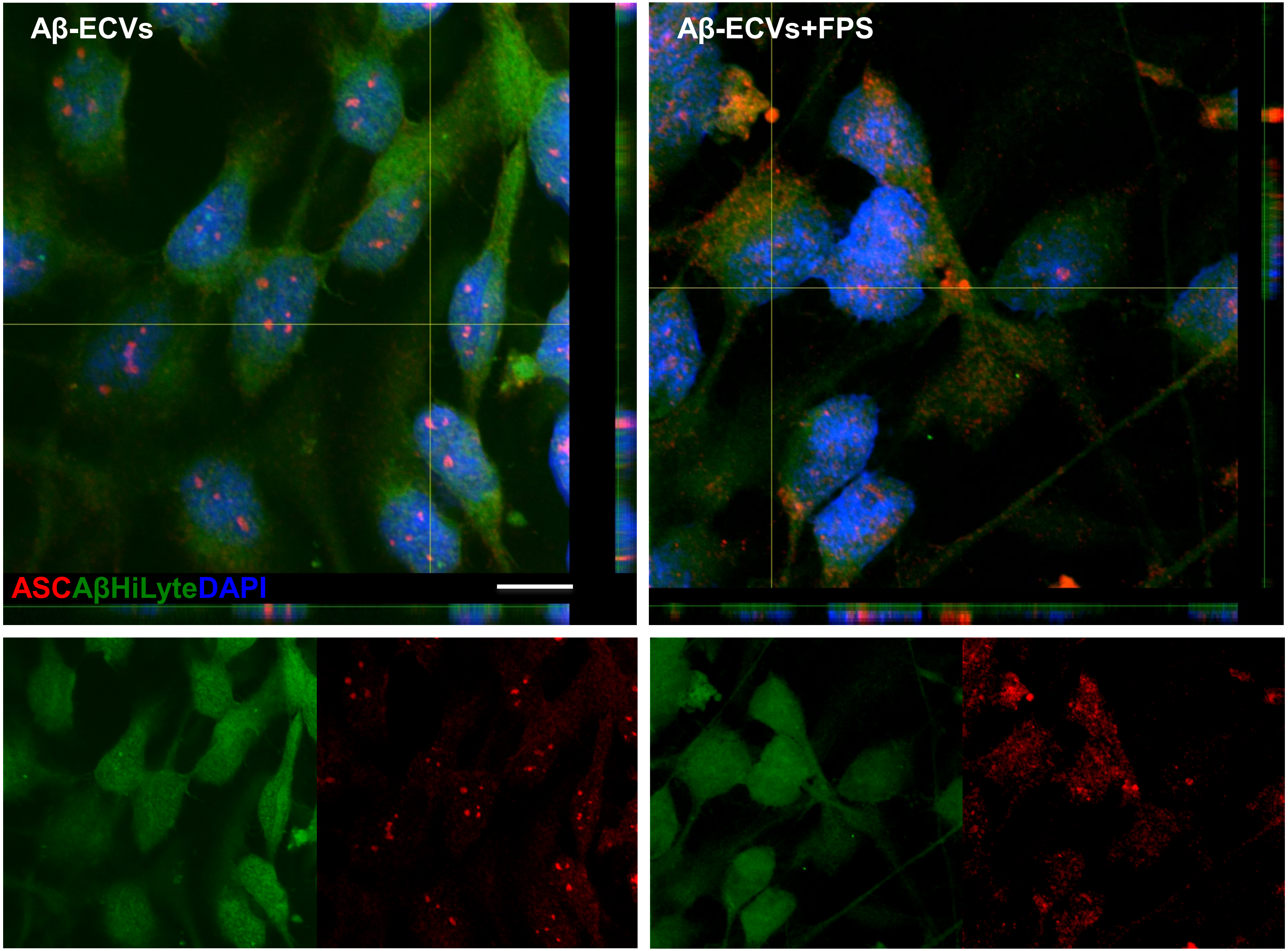

B

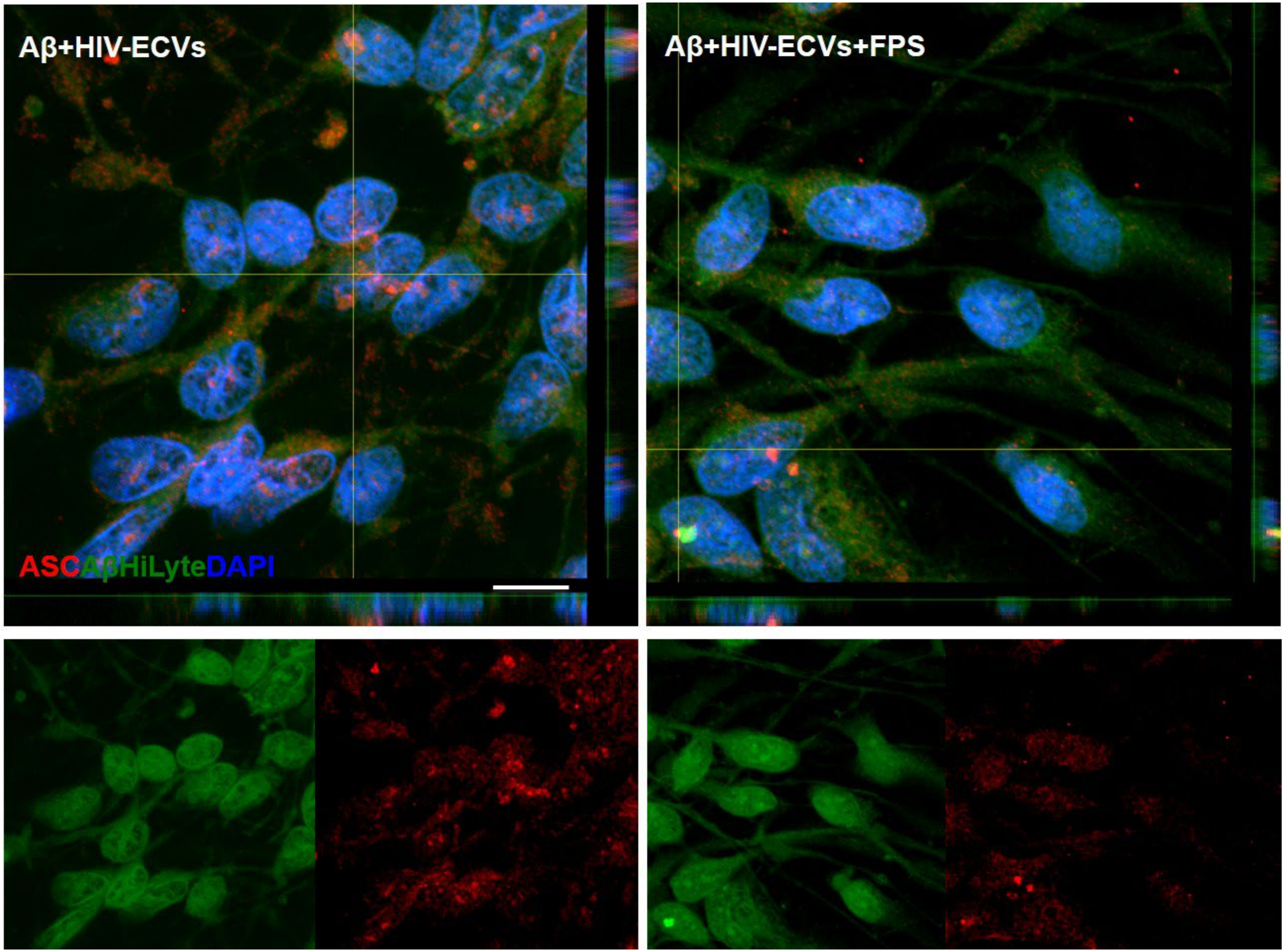

Figure S4. Impact of HBMEC-derived ECVs on colocalization of NLRP3 and ASC with Aβ in NPCs. Related to **Figure 4**. HBMEC were treated with HIV and/or Aβ and ECVs were isolated as in **Figure S2**; however, Aβ (1-40) HiLyte was used instead of non-florescent Aβ (1-40). Then, human NPCs were exposed to HBMEC-derived ECVs for 24 h, with selected cultures additionally treated with 500 nM FPS-ZM1 (FPS) as in **Figure S3**. A) NLRP3 and B) ASC immunoreactivity is shown in red. DAPI staining (blue) visualizes the NPC nuclei. Transferred Aβ (1-40) HiLyte from brain endothelial ECVs is visualized in green. The combined z-stack images are representative from three experiments. Scale bar: 10 µm.

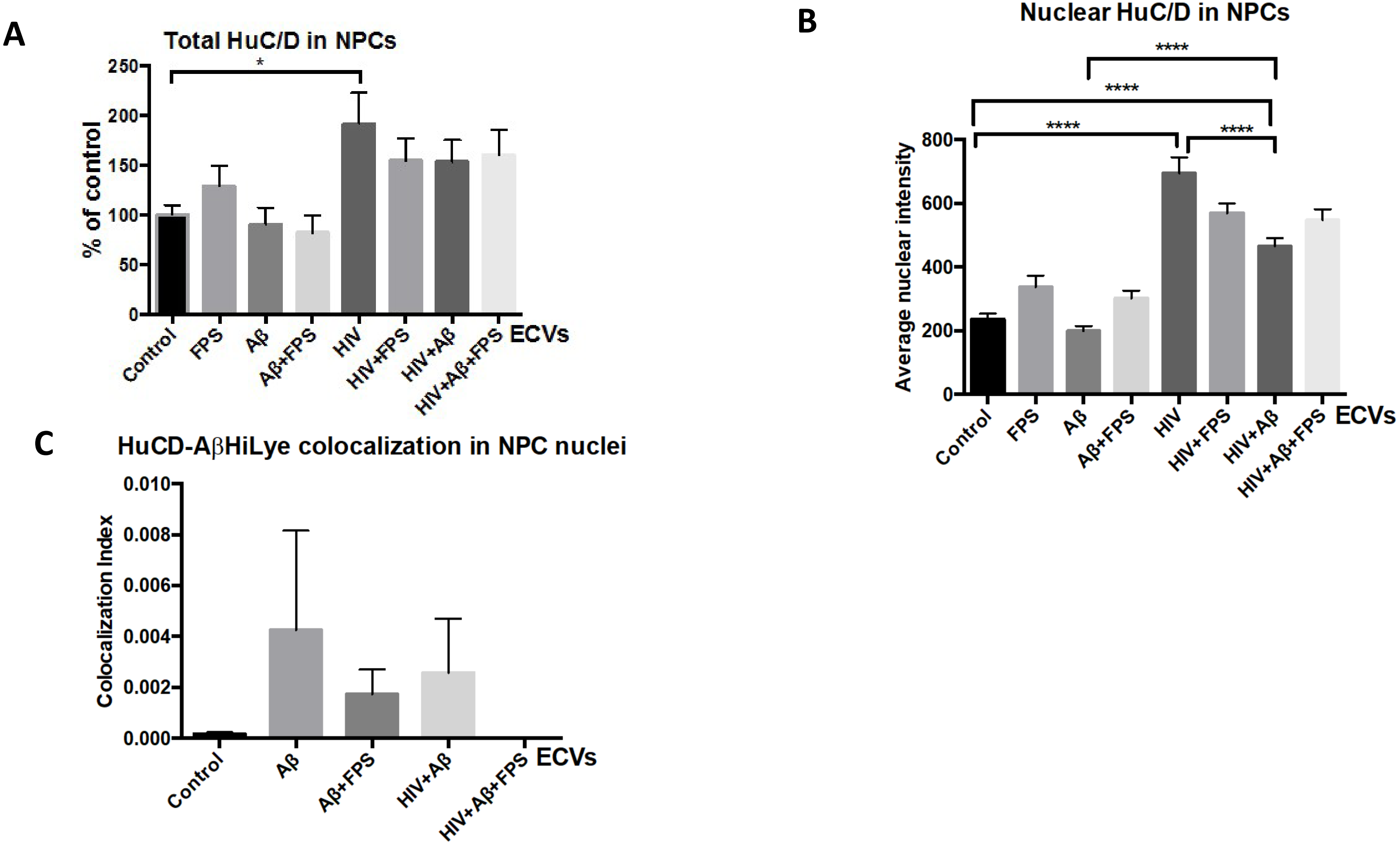

Figure S5. Impact of ECV-mediated Aβ transfer on NPC differentiation as measured by HuC/D. HBMEC were exposed to HIV (30 ng/ml) and/or 100 nM Aβ (1-40) HiLyte for 48 h, followed by isolation of ECVs from the cell culture media. NPC were differentiated for 3 days in the presence of HBMEC-derived ECVs. At the beginning of differentiation, selected NPC cultures were pretreated with 500 nM FPS-ZM1 (FPS) for 2 h followed by cotreatment with the isolated ECVs. At the end of the 3-day differentiation, the neuronal marker Hu C/D was assessed by confocal microscopy. A) Total intensity of Hu C/D immunoreactivity as quantified from the confocal images. B) Intensity of nuclear Hu C/D immunoreactivity as quantified from random nuclear areas. C) Quantification of Hu C/D colocalization with Aβ (1-40) HiLyte in random nuclear areas. Values are mean ± SEM, n=28 (total); n=179-180 (nuclear); n=120 (colocalization). Statistically significant at $^{*}p<0.05$, $^{****}p<0.0001$.

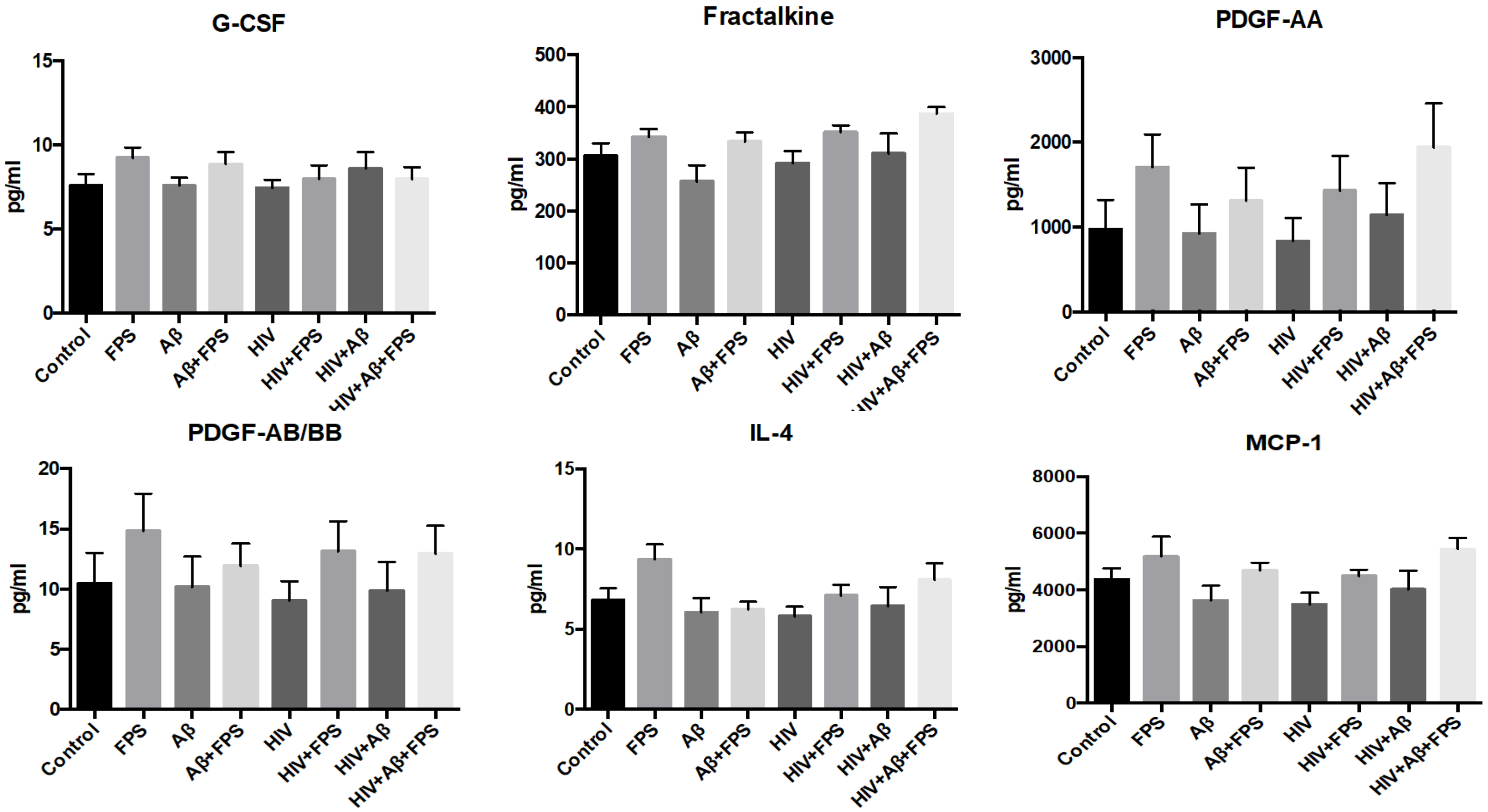

Figure S6. Cytokine/chemokine panel after NPC differentiation. NPC were treated and differentiated as in **Figure 5**. At the end of the 3-day differentiation, G-CSF, IL-4, MCP-1, Fractalkine, PDGF-AA, PDGF-AB/BB levels were measured by Luminex MagPix assay from the cell culture media. Values are mean ± SEM, n=5-8. NS, not statistically significant
